# Atypical AT Skew in Firmicute Genomes Results from Selection and Not from Mutation

**DOI:** 10.1371/journal.pgen.1002283

**Published:** 2011-09-15

**Authors:** Catherine A. Charneski, Frank Honti, Josephine M. Bryant, Laurence D. Hurst, Edward J. Feil

**Affiliations:** 1 Department of Biology and Biochemistry, University of Bath, Bath, United Kingdom; 2 Medical Research Council Functional Genomics Unit, Department of Physiology, Anatomy, and Genetics, University of Oxford, Oxford, United Kingdom; 3 The Wellcome Trust Sanger Institute, Wellcome Trust Genome Campus, Cambridge, United Kingdom; Stanford University, United States of America

## Abstract

The second parity rule states that, if there is no bias in mutation or selection, then within each strand of DNA complementary bases are present at approximately equal frequencies. In bacteria, however, there is commonly an excess of G (over C) and, to a lesser extent, T (over A) in the replicatory leading strand. The low G+C Firmicutes, such as *Staphylococcus aureus*, are unusual in displaying an excess of A over T on the leading strand. As mutation has been established as a major force in the generation of such skews across various bacterial taxa, this anomaly has been assumed to reflect unusual mutation biases in Firmicute genomes. Here we show that this is not the case and that mutation bias does not explain the atypical AT skew seen in *S. aureus*. First, recently arisen intergenic SNPs predict the classical replication-derived equilibrium enrichment of T relative to A, contrary to what is observed. Second, sites predicted to be under weak purifying selection display only weak AT skew. Third, AT skew is primarily associated with largely non-synonymous first and second codon sites and is seen with respect to their sense direction, not which replicating strand they lie on. The atypical AT skew we show to be a consequence of the strong bias for genes to be co-oriented with the replicating fork, coupled with the selective avoidance of both stop codons and costly amino acids, which tend to have T-rich codons. That intergenic sequence has more A than T, while at mutational equilibrium a preponderance of T is expected, points to a possible further unresolved selective source of skew.

## Author Summary

When considering a single strand of DNA, it is not necessarily the case that the frequency of each base should equal its complementary partner, such that A = T and G = C. For the leading strand, it is typically the case that Gs are more common than Cs, and Ts more common than As. This bias is widely thought to arise due to different mutational biases during replication. The Firmicutes exhibit an atypical preference for A over T on the leading strand, and here we show that selection, rather than mutation, can explain this exception. For those bases within coding regions, selection acts to inflate the frequency of A over T in order to avoid stop codons and to use metabolically cheap amino acids. Because genes are not orientated randomly, this manifests as an overall enrichment of A on the leading strand. Furthermore, a direct examination of mutational patterns is inconsistent with the observed enrichment of As. Curiously, our data also point to an unresolved source of selection on synonymous and intergenic sites, which are widely assumed to be neutral.

## Introduction

Skews in nucleotide usage (compositional asymmetries) are of interest as they provide a window into fundamental processes operating within genomes. Under conditions of equal mutation bias and random gene orientation, the two complementary strands of a bacterial chromosome should be subject to the same sets of substitutions, and hence each should contain approximately equal amounts of a given base and its complement [1]. This condition, where A∼T and C∼G within a given strand, is known as the second parity rule and represents a null expectation of sequence evolution. The division of the replication fork into leading and lagging strands, however, has shaped bacterial sequence evolution contrary to this null, as each strand generally possesses an excess of one nucleotide over its complementary base (called GC and AT skews). Within bacterial genomes, nucleotide skews normally manifest as a richness of G over C and (with a lesser magnitude) T over A on the replicatory leading strand [2]–[5].

These genomic skews indicate some force, be it mutation or selection, is biasing substitutions between the two replicating strands. While it is acknowledged that, in theory, selection for genes to reside in the leading strand coupled with preferences for particular amino acids could result in chromosome-wide skews [2], [5]–[8], such a role for selection in generating large-scale compositional bias remains largely hypothetical and undescribed. Instead mutational biases between the two replicating strands are generally invoked as the cause of nucleotide skew [3], [8], [9]. Mutational differences between transcribed and non-transcribed strands have also been considered [10], [11], and these explanations incorporate a selective element as they require asymmetrically distributed genes between the replicating strands.

It has been argued that strand-specific mutation biases might result from the different amounts of time spent by each strand exposed in the single-stranded state during continuous or discontinuous DNA replication. While cytosine deamination (C→T) in particular was long suspected to play a major role in creating the excess of G and T in the leading strand, it has been shown that similar compositional skews can result from a variety of mutational scenarios [12]. The observation that GC skews tend to be stronger than AT skews also points to contributions from multiple mutation types. As would be expected if they are primarily mutational in origin, detected skews are generally higher in nearly neutral sites such as intergenic regions and fourfold degenerate sites [2], [3], [10].

*Staphylococcus aureus* is an unusual case in that, like other Firmicutes, it displays an excess of A over T in the leading strand, or positive AT skew given as (A–T)/(A+T) [13]. Why does this AT skew run counter to that observed in most bacteria? One possibility is that unique selective processes might be avoiding T and preferring A in the leading strand. Genes predominately lie in the leading strand in *S. aureus*, a feature of bacterial chromosomes posited to result from selection to minimize impacts between DNA and RNA polymerases [14] (although the relevance of this mechanism remains unclear). Any pressure to underuse codons rich in T could then result in AT skews simply due to the differential coding content of these two strands. Gene orientation bias is particularly enhanced in low G+C Firmicutes, potentially on account of the replication fork asymmetry induced by the possession of separate α subunits for synthesis of the leading and lagging strands [15], [16]. Alternatively, *S. aureus* might display a mutational bias which produces AT skew opposite that of most other bacteria, pushing up A over T in the leading strand. Indeed, it was recently suggested that the DNA polymerase-α subunit that replicates the leading strand also determines the direction of AT skew [16]. However, this finding was not repeated in a subsequent study and a direct mutational effect on AT skew resulting from α-subunit possession was called into question [11].

Here we investigate whether mutation or selection best explains the unusual AT skew in *S. aureus*. Dividing the chromosome into coding and non-coding positions allowed us to assess whether skew is strongest in those sites which should be under weaker purifying selection, such as intergenic and fourfold degenerate sites, or whether skew is most prevalent in non-synonymous sites which are constrained by the need to code for amino acids. Moreover we make use of newly described, high resolution genome-wide SNP data representing a single widespread clone of methicillin-resistant *Staphylococcus aureus* (MRSA) [17]. As these isolates have diverged from a very recent common ancestor, over a period of 4-5 decades, the data provide an opportunity to infer mutational patterns in *S. aureus* and contrast AT skews expected under mutational equilibrium to the AT skews observed. Importantly, the false positive rate of SNP calling in these genomes is benchmarked to be less than 1 SNP per genome (Julian Parkhill, personal communication), making these an unprecedentedly high quality resource.

## Results

### AT skew in *S. aureus* is unusual

**Figure 1:**
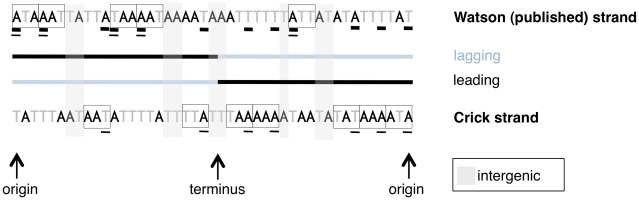
A schematic of sites used for different types of analyses in this paper. A mock linearized *S. aureus* genome is shown, with codons on the strand from which they are transcribed shown in boxes. Note coding content is over-represented on the leading strand. Only first codon positions are considered for the sake of simplicity. The results presented in Figure 2, Figure 3, and Figure 5 consider coding sites underlined with a thick line, meaning the identities of the nucleotides are all taken from the published strand in dedicated coding sites, regardless of the sense direction of the gene. Thus Figure 2 and Figure 3 do not explicitly distinguish between leading and lagging AT skews, but give an averaged picture of skews along both strands of the genome. All other analyses of AT skew in coding sites in this paper consider the sites underlined with a thin line. The identities of these nucleotides are all in the sense direction of the gene, i.e. that nucleotide which appears within the transcript, and may be easily divided into groups according to whether they are encoded on the leading or lagging strand. The analysis of intergenic regions is simpler in concept as intergenic sites clearly divide into either leading or lagging.

As some of our results focus on coding sites within a single DNA molecule, the published strand, whereas others utilize coding sites in the sense direction on either the leading or lagging strands, we have provided a schematic to illustrate which sites are being considered in different types of analyses (Figure 1).

**Figure 2:**
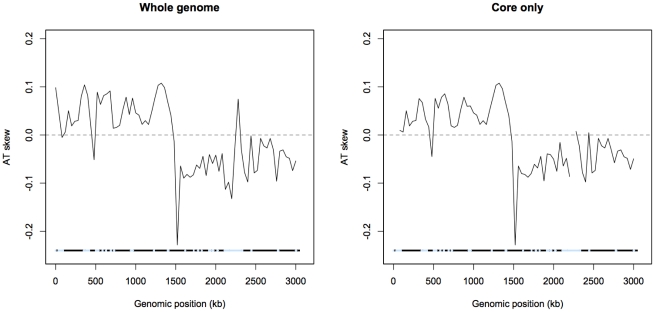
AT skew in *S. aureus*. Skew calculated in 40 kb non-overlapping windows is shown with respect to the published strand. The origin of replication is near 0 kb and the terminus of replication is expected to coincide at the midpoint where the skew changes direction. Excluding non-core (denoted by blue regions) eliminates an A-rich peak at approximately 2,300 kb into the genome.

A plot of AT skew on the published strand in non-overlapping windows confirms that AT skew in *S. aureus* is unlike that of most bacteria as it is positive in the first half of the published strand (Figure 2), as previously described [13]. Considering only the core (vertically transmitted) without non-core (laterally transferred) regions eliminates irregularities in the AT skew which may arise from the importation of sequences which previously resided on an oppositely-skewed strand (Figure 2). For this reason the core genome only was considered in the rest of our analysis.

### AT skew in *S. aureus* is not primarily mutational

Three lines of evidence argue against mutation as the cause of AT skew in *S. aureus*:

#### 1) Evidence from comparison of weakly and more strongly selected sites

**Figure 3:**
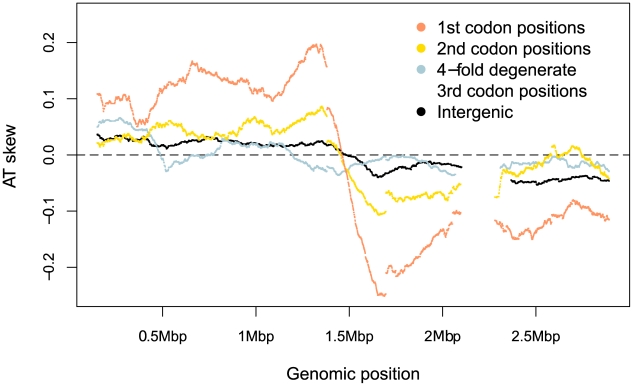
*S. aureus* AT skews in different sites along the genome. Skew was calculated with respect to the published strand using overlapping windows of 300kb and 1 kb steps where a data point was plotted if the number of bases in a window reached at least 30,000. Codon positions were demarcated along the published (Watson) strand and skews in these positions were plotted using the sequence in this single DNA molecule without respect to whether the gene is encoded on the leading or lagging strand.

If skew were primarily mutational, we should expect to see the strongest skew associated with sites which are under comparatively weak selective constraint. Separating the genomic skew into different sites relative to the published strand reveals there is, at best, only a small mutagenic contribution to AT skew evident in weakly-selected intergenic regions and fourfold degenerate sites, where mutations are synonymous (Figure 3, Table 1). Instead the greatest AT skew, by an order of magnitude, is seen in first and second codon positions (Figure 3, Table 1), sites which are largely non-synonymous and should be more buffered against the effects of mutational biases.

**Table 1:** A quantification of *S. aureus* AT skews.

Site	Leading AT skew	Lagging AT skew	P
1st positions	0.2403	0.2081	<0.0001
2nd positions	0.0717	0.0245	<0.0001
4-fold degenerate sites	0.0199	-0.0033	<0.0001
Intergenic (intra-operonic)	0.0021	-0.0193	0.280
Intergenic (ex-operonic)	0.0276	-0.0276	<0.0001

AT skew values in coding positions in this table are given with respect to the protein-coding sense direction of a gene and are differentiated into whether they lie on the leading or lagging strand (see Figure 1). While ex-operonic skews are calculated from all available ex-operonic sequence, intra-operonic skews are calculated only for the strand on which the surrounding genes are transcribed. P was calculated as (*r*+1)/(*n*+1), where *r* is the number of simulated genomes resulting in a difference in AT skew values between the leading and lagging strands equal to or greater in magnitude than that observed in the reference (TW20) genome, and *n* is the total number of simulations performed (10,000).

That fourfold degenerate sites show only a small contribution to overall AT bias provides no evidence for transcription-associated mutation as the prime cause of skew. Even in the case that codon usage bias is acting to alter the transcriptional effects on AT skew in fourfold sites, we still observe that intra-operonic intergenic regions, which are putatively transcribed, display a very weak AT skew similar to that seen in ex-operonic intergenic regions and fourfold sites (Table 1). We thus conclude that if there is a transcriptional mutation bias affecting AT skew, it is rather slight. Indeed, if anything, the lower intra-operonic leading-strand AT skew values relative to ex-operonic leading-strand AT skew is consistent with a transcriptional pressure towards T (over A).

**Figure 4:**
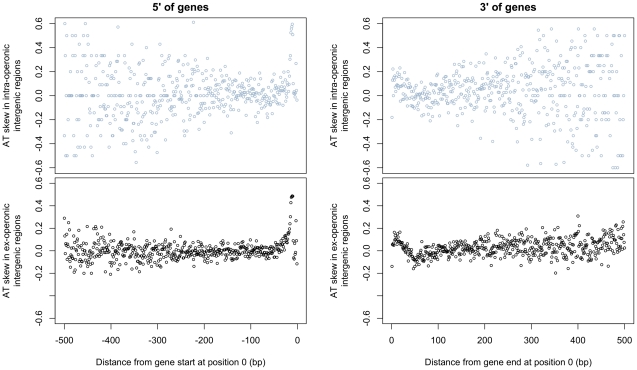
AT skew displays local abnormalities at intergene boundaries but grows neither A nor T rich at increasingly distant positions from gene starts and ends. AT skew at each position was calculated from the nucleotide content measured across all intra- or ex-operonic intergenic regions at that position relative to the gene start or end as appropriate. All intergenic regions were considered in the direction of transcription of the relevant gene, and similar results are obtained when only UTRs of leading strand genes are considered (latter not shown). The effects on AT skew are similar between ex-operonic and intra-operonic intergenic regions for regions both 5′ and 3′ of genes, with more noise apparent at further distances in the intra-operonic regions due to increasingly smaller sample sizes. *5′ of genes*. With increasing distance 5′ of gene starts, AT skew first increases before it decreases. *3*′ *of genes.* Starting at gene ends, AT skew becomes more positive before it decreases again, becoming briefly negative before levelling out.

Additionally, if transcriptional mutation were the cause of atypical AT skew, we should expect to observe a decrease in skew with increasing distance from gene boundaries. This effect would due to the transcription of UTRs of varying lengths by RNA polymerase, with transcription-induced skew decreasing away from gene boundaries as the contribution of increasingly longer UTRs to intergenic regions declines. We would also expect to observe increased AT skew in intra-operonic intergenic sites, which should be more prone to transcriptional effects. Figure 4 shows the patterns of AT skew with increasing distance from 5′ (upstream) and 3′ (downstream) gene boundaries in intra-operonic and ex-operonic sites. Although we note striking deviations in AT skew at both boundaries [see also 18,19], these patterns are not monotonic and therefore cannot be explained by transcriptional effects. Instead, we consider it likely that these deviations correspond to translational initiation and termination signals, and similar effects are observed in other species (Figure S1). Furthermore, the patterns are very similar for intra-operonic and ex-operonic intergenic regions, the increased scatter in the former sites reflecting a smaller sample size (Figure 4). On account of these deviations in skew at the boundaries of intergenic regions, 60 bp were removed from each end of intergenic regions for all subsequent analyses.

#### 2) Evidence from rare SNPs

From examination of rare and putatively weakly-selected SNPs, which should better reflect mutational pressures, we can infer the mutational profile in *S. aureus* and the corresponding nucleotide frequencies expected at mutational equilibrium and hence the expected skew at mutational equilibrium (Table 2). We considered that not only might selection on codon usage be biasing observed SNPs in fourfold degenerate sites, but that fourfold sites are biased in terms of the possible nucleotides that may precede them in the second codon position and thus may give a distorted representation of mutational processes due to over- or under-represented dinucleotide effects. Instead we consider that the mutational profile in intergenic regions would best reflect the AT skew expected to result from replication-associated mutation. Under the premise that intra-operonic intergenic regions are more likely to be subject to transcriptional pressures (e.g. for regulation of translational coupling [20]), we limited our analysis of replication-associated mutation to ex-operonic intergenic regions, intergenic being defined as more than 60 bp from either end of a gene and with a 500 bp maximum length cutoff.

**Table 2:** Relative mutation rates of nucleotide i to j per site i for ex-operonic intergenic sites were calculated from singleton SNPs for the two replicatory strands.

		from A	T	C	G	Equilibrium frequency	Equilibrium AT skew
**Leading**	to **A**	-	1.79965E-04	4.37222E-04	6.39046E-04	0.2257	-0.4176 (-0.6792, -0.1522)
	**T**	2.34002E-04	-	1.67602E-03	1.17158E-03	0.5493	
	**C**	6.38189E-05	4.72409E-04	-	5.32538E-05	0.1319	
	**G**	6.59462E-04	4.49913E-05	0.00	-	0.0931	
**Lagging**	to **A**	-	2.34003E-04	1.17158E-03	1.67602E-03	0.5493	0.4176 (0.6792, 0.1522)
	**T**	1.79965E-04	-	6.39046E-04	4.37222E-04	0.2257	
	**C**	4.49913E-05	6.59462E-04	-	0.00	0.0931	
	**G**	4.72409E-04	6.38189E-05	5.32538E-05	-	0.1319	

Relative rates were derived from the following leading strand ex-operonic SNP counts, where XY represents a change from nucleotide X to Y: AG 31 GA 12 CG 0 GC 1 GT 22 TA 8 TC 21 TG 2 AC 3 CA 6 AT 11 CT 23. Nucleotide frequencies at compositional equilibrium were derived from the relative mutation rates. Leading and lagging equilibrium AT skews were calculated from equilibrium A and T frequencies. 95% bootstrap intervals are shown in parentheses.

Importantly, ex-operonic intergenic SNPs indicate that single base mutations acting alone would lead to a strong excess of T over A in the intergenic leading strand at compositional equilibrium (Table 2). Bootstrapping the *S. aureus* data (see Methods) supports the view that the expected compositional equilibrium is negative (Table 2). Additionally, the intergenic equilibrium AT skew expected for a second Gram positive Firmicute which also displays an unusual (positive) AT skew in the leading strand, *Bacillus anthracis*, is negative (Table S1). In this case the appropriateness of the SNPs for this sort of analysis is not so easily demonstrated as the sequencing was performed by multiple groups and in several instances we cannot find statements of the quality of the sequencing. However, in a further non-Firmicute for which high quality recent SNPs are available, the Gram negative *Salmonella enterica str. typhi*, we again find that the SNP profile predicts the typical (negative) leading AT skew (Table S1), suggesting the predicted neutral equilibrium T>A bias in the leading strand of *S. aureus* is not unusual. In the case of both *B. anthracis* and *S. typhi*, as the sample size of recent SNPs is relatively small, less confidence can be given to the equilibrium values than in the case of *S. aureus* (Table S1).

In addition, if selection were operating on intergenic SNPs we should expect that older SNPs will predict an equilibrium skew closer to that observed, as selection will have had longer to operate on older SNPs, potentially removing the weakly deleterious ones. To test this we examine 54 SNPs that were found in two, three or four *S. aureus* isolates. We find that the predicted equilibrium AT skew obtained from the 54 ex-operonic SNPs present in multiple isolates (equilibrium AT skew  = 0.4757, 95% bootstrap interval: 0.0763, 0.8087) is of completely different sign than that obtained using 140 ex-operonic singletons (-0.4176, 95% bootstrap interval: -0.6792, -0.1522). A randomization was used to put a significance level on whether these values are significantly different. The singleton SNPs and those SNPs present in 2, 3, or 4 isolates were combined into one large group. From this group SNPs were sampled with replacement and randomly allocated to either the singleton SNP group (140 SNPs) or the 2, 3, or 4 isolate group (54 SNPs). These singleton and 2, 3, 4 strain groups were randomly simulated 2000 times, and for each simulation the equilibrium AT skew was calculated for the two random groups and the difference between the two equilibria determined. P was calculated as (*r*+1)/(*n*+1) where *r* is the number of simulations which produced a difference between the singleton and 2, 3, 4 group greater than or equal in magnitude to that observed and *n* is the number of randomizations performed. This test indicates these two groups of SNPs are different as regards the expected AT skew (P = 0.0065). The assumption that older SNPs are more prone to selection is supported by analysis of dN/dS ratios for SNPs in genes: the average dN/dS over all pairwise genomic comparisons is 0.69, but that for genomes diverged by fewer than 10 SNPs approaches 1 [21], indicating that recent SNPs have not yet had time to be purged [22] and better reflect the mutational profile.

The above results support the view that the AT skew cannot be explained by mutation bias, but also have broader implications for inferring mutational patterns. The striking difference in the mutational profiles inferred from singleton SNPs and from those SNPs present in multiple isolates points to a class of mutation which, though non-lethal (and therefore observable), is sufficiently deleterious to be purged very rapidly by selection. Thus, in order to make the most reliable inferences of mutational profiles (and hence predicted equilibria) it is necessary to consider extremely recently-emerged singleton SNPs between very closely related genomes, with the caveat that the low number of SNPs per clone be offset by large samples of genomes.

The reliability of sequence data is of the utmost importance when using singletons, and such SNPs have been avoided in the past owing to the possibility of sequencing errors [23]–[27]. However, we are confident that false positive SNPs cannot account for our results. With a remarkable benchmarked false-positive rate of no more than 1 per genome (Julian Parkhill, personal communication), the maximum number of false positive ex-operonic SNPs in our analysis is (the fraction of intergenic sequence which is ex-operonic) * (the error rate of 1 base per genome) * (the number of genomes used)  = (125042/3043210)*1*62≈2.55, or about 3. We used randomizations to assess the effect that these potential miscalled SNPs would have on our AT skew calculations. For each simulation, 3 (hypothetical false positives) of the 140 ex-operonic SNPs were removed at random and the equilibrium AT skew resulting from the remaining SNPs was calculated, with 1,000 simulations performed in total. All of the resulting leading strand equilibrium AT skew values are negative and fall between −0.3830 and −0.4910, indicating false positives are not affecting our inference of the sign of the equilibrium AT skew. For these reasons we contend that an analysis of very recent singletons is the best reflection of the mutational profile. To the best of our knowledge the data set we examine is the only one of high enough quality for recently diverged (post 1960 [17]) lineages.

It is possible that that even the intergenic singleton SNPs have been affected by selection and are hence not an unbiased reflection of the mutation profile. If so, the difference between the true mutational equilibrium and the observed composition would only be greater, making our current analysis conservative. Thus replication-associated mutation cannot account for either the leading strand excess of A over T that is observed either in *S. aureus* intergenic regions (Table 1) or along the entire chromosome (Figure 3).

#### 3) Evidence for skew being independent of replicatory strand

**Figure 5:**
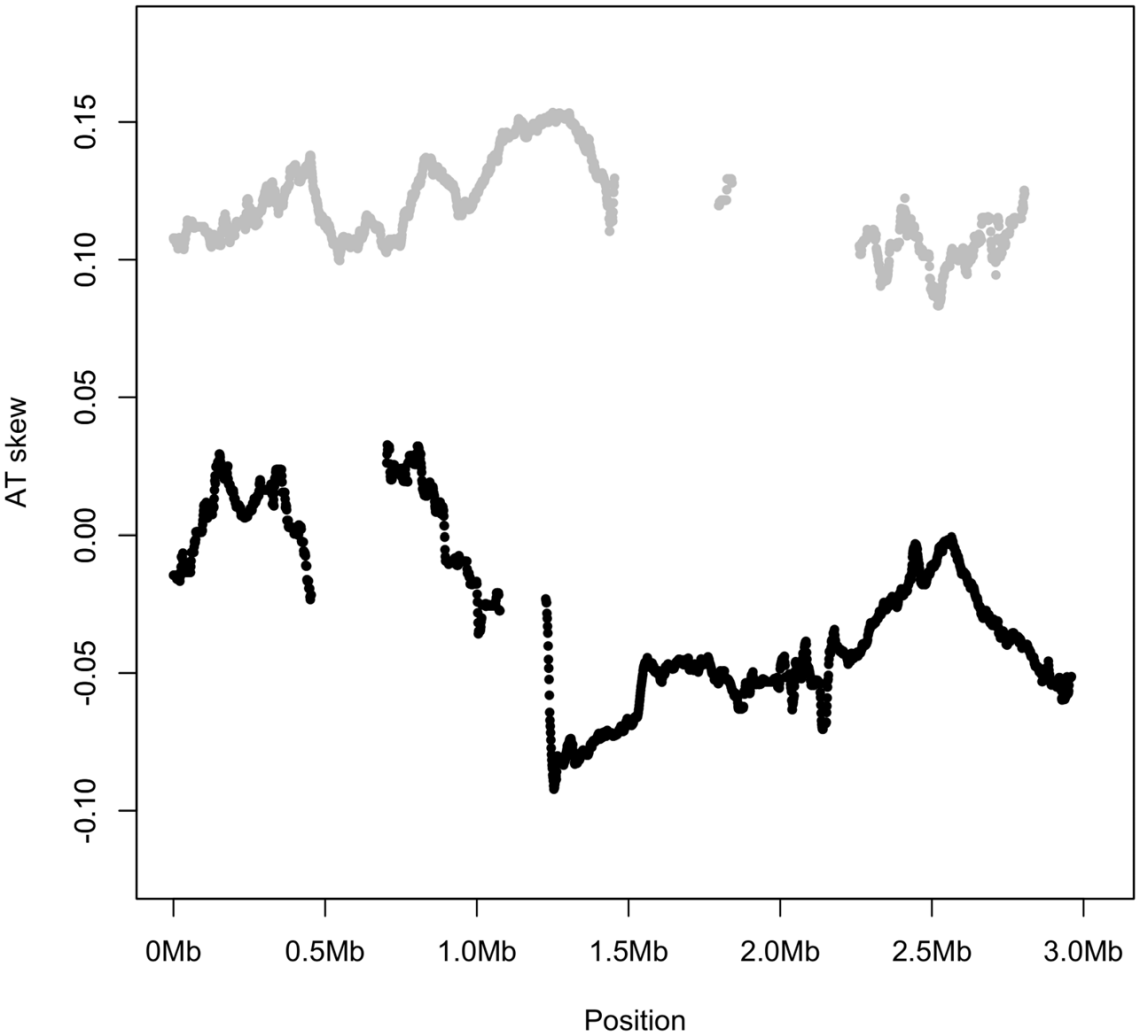
AT skew is similar along the entire length of genome for all noncomplementary genes (gray), while all complementary genes (black) display a similar AT skew. Genic AT skew was calculated with respect to the published strand, using overlapping windows of 300 kb and 1 kb steps where a data point was plotted if the number of bases in protein-coding genes in a window reached at least 30,000. Thus the skew shown for the noncomplementary genes (gray) corresponds to their coding sense direction whereas the skew shown for complementary genes (black) corresponds to their coding antisense direction. Skews were plotted this way to visually distinguish between different segments of the replicatory strands. If AT skew were primarily induced by replication, leading strand genes (the first half of the gray strand and the second half of the black strand) should show similar skews, and lagging strand genes should show roughly uniform skews opposite in sign to the leading strand genic skews. However this is not the case and *S. aureus* genes show AT skew corresponding to the direction in which they are transcribed, or the direction in which their amino acids are encoded.

First and second codon sites, where the greatest AT skew is found, have signs of skew corresponding to the direction in which they are transcribed, irrespective of which replicating strand they lie on (Figure 5). This suggests replication-induced mutation is not contributing to the observed AT skews since such mutation would be expected to oppositely impact the magnitude of skew in the leading and lagging segments of a single DNA molecule.

These results indicate that the strong AT skew seen on the leading strand is dominantly owing to a strong strand bias, with the great majority of genes co-oriented with the replicatory fork (the leading strand contains 78% of the core coding content in *S. aureus*) and an abundance of A over T in coding sequence.

### Avoidance of stop codons leads to AT skew at first codon positions

Having established that mutation is not the primary cause of the observed AT strand bias, we sought to determine what selective forces might be responsible. The principle challenge appears to be to explain why AT skew is so profound at first sites in codons, even when compared with codon second sites. As stop codons start with T and cannot feature within the coding sequence, their avoidance provides a potential component of the unusual first site AT skew.

While we are able to make an *a priori* assumption regarding stop codon usage (since, by definition, they cannot be included within the body of a gene), we have no prior expectation concerning amino acid usage. It was therefore necessary to measure the AT skew resulting from biased gene distribution in *S. aureus* in the absence of selection on amino acid-encoding codons. To this end we simulated coding sequences preserving the discrepancy in coding content between the two replicating strands of *S. aureus*. For each simulation, the same number of codons as seen in a given replicatory strand were reconstructed based on the intergenic nucleotide frequencies within that strand, but with the caveat that stop codons were not permitted. Intergenic base frequencies were used to derive codons in order to control for any effects of the baseline nucleotide content as well as any mutational contribution to coding content within the chromosome. AT skew was then calculated in first and second sites for each of the 10,000 randomized coding sequences. These simulations quantified the AT skew expected to result purely from the avoidance of stop codons, indicating that randomized coding sequences display significant AT skew in first positions (Table 3). Thus, we would expect a lack of stop codons to contribute significant AT skew in first positions given such a discrepancy in coding content between the two replicating strands, even with a complete lack of selection on amino acid content. However, the magnitude of the effect owing to stop codon avoidance is unable to explain the full magnitude of the skew that we observe.

**Table 3:** Simulated AT skew (that which can be accounted for by avoidance of stops in coding frames) in first and second codon positions contrasted with skews observed in *S. aureus* among all protein-coding genes.

Strand	Sites	Observed AT skew (TW20)	Mean simulated AT skew	P
**Leading**	1st positions	0.2403	0.1767±0.0016	<0.0001
	2nd positions	0.0717	−0.0731±0.0016	<0.0001
**Lagging**	1st positions	0.2081	0.0850±0.0030	<0.0001
	2nd positions	0.0245	−0.1250±0.0029	<0.0001

Both simulated and observed skews are given in the sense direction.

### Selective pressure for cost-effective amino acids leads to AT skew

**Figure 6:**
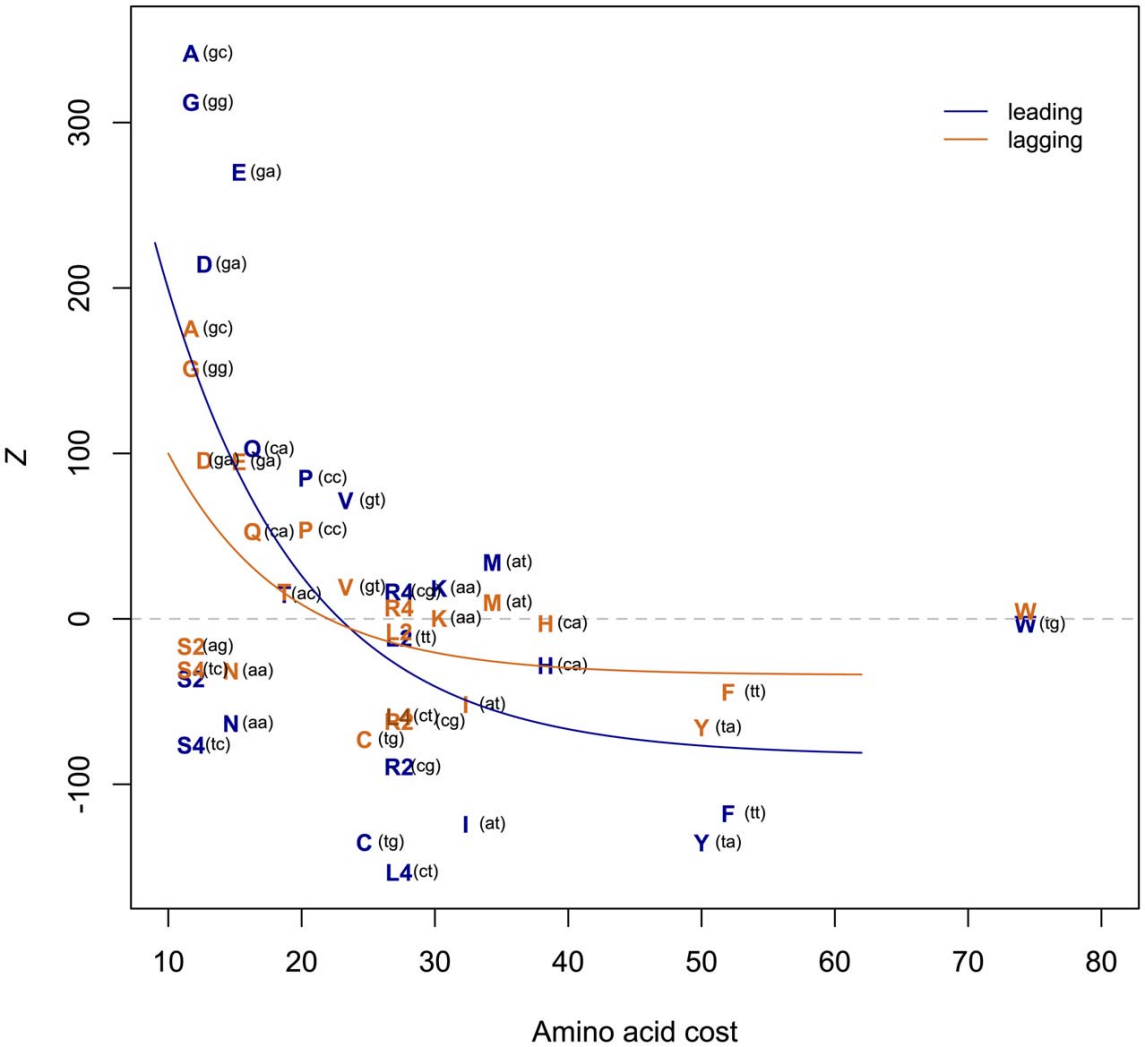
T-rich codons are under-represented in *S. aureus*. A positive *Z* represents over-usage, a negative *Z* under-usage. The negative correlation between *Z* and amino acid cost implies avoidance of costly (T-rich) amino acids, with the effect stronger on the leading strand (leading Spearman's rho-0.466, one-sided P = 0.013; lagging rho -0.468, P = 0.012). The first two codon positions of each amino acid are indicated in parentheses. Amino acid costs from{Akashi, 2002 #65} Akashi and Gojobori 2002 [29].

To explain the residual AT skew at first sites and all of the AT skew at second sites left unexplained by the avoidance of stop codons in reading frames, we investigated the possibility of further selection within coding sequences to decrease T. The mean codon usage in the randomized coding sequences (where codons are drawn randomly in proportion to the intergenic nucleotide frequencies within the same replicating strand) represents a null expectation of codon usage in the absence of any selection on amino acid content. Comparing this null with the observed codon usage in the TW20 chromosome allows for direct quantification of the over- or under-usage of a given codon (*Z*, see Methods). A positive *Z-*score for a given amino acid indicates that amino acid is more commonly used within the *S. aureus* genome than would be expected according to our null model of codon usage, while a negative *Z* indicates that amino acid is under-used. Such an approach reveals that T-rich codons are in fact highly under-represented in the *S. aureus* chromosome, with an enhanced avoidance of T in the gene-rich leading strand (Figure 6). What selective force could account for such a paucity of T in first and, to a lesser extent, second sites? We hypothesize that it might reflect unusual features of the amino acids that start with T.

T-starting amino acids mostly belong to the shikimate pathway and are thought to have been those most recently added to the genetic code [28]. As late-added amino acids tend to be expensive to manufacture [29] and shikimates in particular tend to have complex chemical structures, might it be that T avoidance reflects nothing more than selection against the use of costly amino acids? We find that there is a significant negative correlation between *Z* and amino acid cost as given in Akashi and Gojobori 2002 [30] (Figure 6), indicating that expensive amino acids are indeed under-used, thus accounting for some of the paucity of T. We have repeated this analysis using alternative cost measures [31] and obtained similar results for six out of eight cost schemas (Figures S2, S3, S4, S5, S6, S7, S8, and Table S2). The two measures (*R_glucose_* and molecular weight) that do not provide significant correlations between *Z* and amino acid cost are perhaps expected to be less revealing: *R_glucose_* correlates neither with previous cost measures nor with amino acid substitution rate, while molecular weight does not take into account metabolic networks relating to amino acid production [31].

### High gene strand bias can also account for atypical AT skew in other Firmicutes

**Figure 7:**
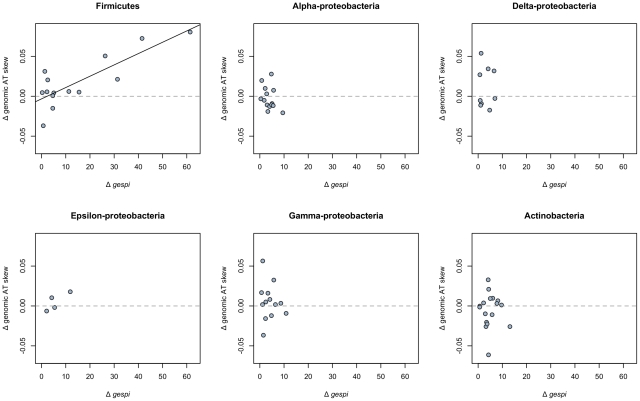
Gene strand bias predicts the extent of positive AT skew in the Firmicutes. Among Firmicutes, an increase in the degree of strand bias (positive Δ*gespi*) between terminal node species results in an increase in genomic AT skew, measured with respect to the leading strand (regression slope P<0.001, r^2^ = 0.65; a one-sided binomial test for association between positive Δ*gespi* and positive Δgenomic AT skew is also significant at P<0.01). Consistent with our model that strand bias dictates the extent of positive AT skew, and therefore no change in *gespi* should not result in a change in AT skew, we cannot reject that the y-intercept goes through 0 (intercept P = 0.628). There is no detectable significant relationship between changes in strand bias and genomic AT skew among members the following phyla (regression slope P values given in parentheses): the Alpha-proteobacteria (P = 0.196), Delta-proteobacteria (P = 0.984), Epsilon-proteobacteria (P = 0.185), Gamma-proteobacteria (P = 0.654), and Actinobacteria (P = 0.87).

We have shown that a lack of stop codons and avoidance of costly amino acids in asymmetrically distributed open reading frames can in large part account for the positive AT skew in *S. aureus*. Could similar mechanisms produce the unusual AT skews seen across other Firmicutes? Using phylogenetically independent contrasts (see Methods), we note that, among Firmicutes, an increase in the degree of gene strandedness from one species to another also results in a proportional increase in the extent of positive AT skew (Figure 7). It is therefore likely that the high degree of gene strand bias similarly explains the atypical patterns of AT skew in other Firmicutes.

As a contrast to the Firmicutes, we performed a similar analysis on phylogenies (Tables S3, S4, S5, S6, S7, S8) of the Gram negative Alpha-proteobacteria [32], Delta-proteobacteria [33], Epsilon-proteobacteria [34], Gamma-proteobacteria [35], as well as the Gram positive Actinobacteria [36]. Although the species sampled from these phyla tend to display typical (negative) genomic AT skews, it is possible that the degree of strand bias within these genomes nevertheless modulates the magnitude of these AT skews due to either avoidance of stop codons or selection on amino acid cost. The additional lineages do not however reveal any pattern of regression of ΔATskew on Δ*gespi*, the latter indicating changes in gene strand bias (Figure 7). This is not unexpected since lack of large values of strand bias between terminal node pairs of the non-Firmicute phyla (resulting in lack of large differences in strand bias) means that the points all scatter around 0.

## Discussion

Recently an interest has emerged in whether certain sites in bacterial chromosomes commonly thought to be nearly neutral are in fact under selection as regards their nucleotide content. Two studies [23], [24] both used SNP profiles to estimate the GC content in possibly neutral sites at mutational equilibrium and showed the observed GC:AT bias greatly differs from that expected under the influence of mutation alone, consistent with previous reports of mutational pressure towards AT in *E. coli* [37]. What these studies were unable to explain, however, was what selective forces might be biasing nucleotide content at third codon and intergenic sites, leaving open the possibility that biased gene conversion and not selection might be acting. Here we also investigate a feature of bacterial chromosomes commonly presumed to be mutational, AT skew, and test whether mutation or selection is responsible for generating the unusual AT skews in *S. aureus*. Not only do we show that the atypical AT skew pattern in *S. aureus* is not due to mutational bias, but we are able to delineate to some degree what mode of selection is occurring (at least in terms of coding sequence), and to what end, in order to explain the observed skew pattern.

We find the mutational effect on AT skew in *S. aureus* (and in another Firmicute, *B. anthracis*), as derived from intergenic SNPs some distance away from coding sequence, to be inconsistent with, and poorly explanatory of, the observed base composition. Fourfold degenerate sites and intergenic regions display little skew, and intergenic SNP profiles do not support a replication-induced mutational origin of AT skew. In addition to fourfold sites, intra-operonic intergenic regions also display very weak AT skews, and hence any transcriptional effect is likely to be weak. Instead our results support a selectionist basis for compositional bias in *S. aureus* in which AT skew, the majority of which is observable at first and second positions in the sense direction, results from selection at both the translational level and on gene position. The avoidance of stop codons and codons encoding costly amino acids accounts for a substantial proportion of the skew in first and second codon positions because the majority of genes are on the leading strand. However, we are unable to accurately quantify the contribution of the avoidance of costly amino-acids, because the cost estimates used [31] are only approximate. Nevertheless, we can describe a relationship between the intensity of selection against costly amino acids and the magnitude of skews (Figures S9, S10, and Table S9). Codons encoding more costly amino acids tend to be AT-rich [29] and we observe that, on average, AT-rich genomes encode more costly amino acids (Figure S9A). However, the average cost of amino acids in AT rich genomes while high, is not as high as expected given the AT pressure (Figure S9B). This we interpret as evidence for more efficient selection against costly amino acids in GC-poor strains, which in turn contributes to a higher AT skew (Figure S10).

We further show a phylogenetically controlled positive association between the extent of gene strand bias and positive genomic AT skew across the Firmicutes, indicating that strand bias is likely responsible in part for the atypical AT skews seen across this phylum. Our failure to detect such a relationship in non-Firmicute phyla may in part be due to a lack of genomes in these phyla with very high strand bias, leaving only increases in strand bias of smaller magnitude to investigate and thus much noisier data sets (Figure 7). The pattern of Δ*gespi* versus ΔATskew observed for the Firmicutes is similar to the patterns observed in other phyla when considering the region 0>*x*<10 (Figure 7), meaning large differences in strand bias between terminal node pairs are required to be able to detect a relationship between the two quantities. This may be why we only see an effect in the Firmicutes, where strand bias is high enough to leave a clear impact upon the magnitudes of genomic AT skews. We conclude that if there is a relationship between *gespi* and AT skew in non-Firmicutes, our method is not sensitive enough to detect it.

Our results leave several mysteries. First, why do species differ in the degree of strand bias and why is it so high in many Firmicutes? These issues remain enigmatic. A simple model supposes that in fast replicating species the chance of DNA and RNA polymerases colliding must be higher than in slow replicating species. There is, however, no correlation between growth rate and gene strand bias [38]. Rather the higher biases are typically found in chromosomes containing two different (possibly strand-dedicated) DNAP α-subunits at the replication fork which may render them more vulnerable to polymerase collisions [15]. It has also been suggested that strand bias reflects gene essentiality rather than the level of expression [39] although, again, this does not explain the unusually high level of strand bias in Firmicute chromosomes.

Further, while both the observed AT skew in non-coding sites and the pattern of SNPs in intergenic sequence cannot explain the skew seen across the leading strands as a whole, the two approaches are also inconsistent with each other. The relative mutation rates calculated from intergenic SNPs indicate that mutation is acting to bias T over A in intergenic sites, which is the typical direction that AT skew takes in most (e.g. many non-Firmicute) bacteria, suggesting less variable skew-related mutational profiles among bacteria than is commonly assumed. However, intergenic sites on the leading strand have a weak bias in the opposite direction. Such a leading strand bias is consistent with leading strand coding sites also showing slightly higher bias than lagging strand coding sites (Table 1).

What could account for the discrepancy between mutational biases and observed base frequencies at putatively neutral sites? One possibility is that these sites are not yet at mutational equilibrium. This could occur if, for example, there were until recently some unannotated small protein coding genes in the “intergene” spacer. These new pseudogenes would take an appreciable time to reach mutational equilibrium and could well leave a trace of A>T skew if they tended to be on the leading strand. However, in this case it is curious that intergenic skew, intra-operonic skew and skew at four-fold degenerate sites all show a weak A>T bias. An alternative is that the weakly positive intergenic AT skews could reflect ongoing selection. One possibility is that there exists unannotated coding sequence, which, if enriched on the leading strand, would contribute a net A>T skew. Neither missing gene model can explain why skew at four fold degenerate sites is of the same magnitude as in putative intergene spacer. In addition, if mutation alone dictated intergenic AT skews, leading intergenic spacers should skew to roughly -0.4 (Table 2) according to our estimates of mutational equilibria. Given that the average leading AT skew in *S. aureus* coding sequence is approximately 0.1 across all three codon positions (Table 1), the vast majority of intergenic spacers would need to be unannotated protein-coding sequence in order for missing genes to be able to explain the observed leading intergenic AT skew of 0.0276 (Table 1), a highly untenable scenario. Removal of the few regions with outlier AT skew values does not substantially impact the intergenic AT skew (Figure S11), suggesting that even if we are missing some genes their contribution to skew cannot explain the overall bias. What exactly is generating weakly positive AT skews in leading intergenic regions remains a mystery, but this analysis adds weight to the growing evidence [see e.g. 19,23,24,37,40] that “neutral” sites in bacterial chromosomes may not be quite so neutral after all.

## Methods

### Sequence

The complete annotated genome of *S. aureus* subsp. *aureus* TW20, accession number FN433596 [41] was downloaded from the EMBL Nucleotide Sequence Database (http://www.ebi.ac.uk/embl/). Data analysis was carried out using Tcl and Perl scripts and statistical analyses were done in R 2.9.0 [42]. Core and non-core regions were delineated as in Harris *et al*. 2010 [17]. Coding sequences labeled as gene remnants or pseudogenes were excluded and both known and putative genes were considered. As we considered intergenic regions to be indicative of mutational pressures, all intergenic regions were subject to a length restriction of 500 bp to decrease the possibility of unannotated genes, and 60 bp were trimmed from each end of all intergenic regions, as these regions display distinct AT skew patterns deviating from that induced by replication alone (see Results and Figure 4). This is most likely explained by ATG initiation context definition (at the 5′ end) and termination sequences (at the 3′ end).

### Defining operons

Distinguishing which intergenic regions lie within operons should help reveal the contribution of transcriptionally-induced mutation to AT skew as such regions should be more likely to be transcribed. The operon structure of *S. aureus* strain MSSA476 [43] was used to deduce operons within strain TW20. Operonic protein-coding and RNA genes in MSSA476 were extracted from NCBI RefSeq NC_002953 (http://www.ncbi.nlm.nih.gov) and were matched via BLAST 2.2.24 against all TW20 protein sequences and gene-encoded RNA sequences respectively. Orthologous operonic genes in TW20 were taken to be those with at least 90 percent identity and an e-value of less than 0.0001. Due to the close evolutionary relationship between the two strains most matches were unambiguous. Matches to pseudogenes and genes in the TW20 non-core chromosome were excluded. TW20 operons were then deduced from these orthologs with the additional caveat that genes within a putative operon be adjacent and transcribed in the same direction. In several cases a gene not contained in an MSSA476 operon was detected inserted into the intra-operonic TW20 intergenic sequence but on the strand opposite to the operonic genes. In these cases the operon was included.

### SNP analysis

To determine whether the observed AT skew deviates from that expected under mutation alone, we require an estimation of nucleotide content, and the resulting AT skew, at mutational equilibrium. 140 singleton SNPs from core ex-operonic intergenic sites at least 60 bp away from a gene boundary were isolated from what is currently the largest SNP dataset for any bacterial species, that comprising 63 *S. aureus* ST239 isolates [17]. In order to check for the possibility that some of these SNPs were called erroneously, we went back to the original read data for each of these 140 SNPs individually. These data revealed an average of 20-fold coverage, a maximum of 46-fold coverage, and a minimum (in 4 SNPs) of 12-fold coverage (Table S10). Furthermore, in 126/140 (90%) of cases, the assigned SNP was consistent in all mapped reads. Of the 14 remaining SNPs, a single inconsistent read was noted in 13 cases, and two inconsistent reads noted in one case. Given a sequencing error rate of 0.5% per sequencing reaction, the maximum probability that any SNP has been assigned by error (that is called consistently in at least 12 reads) is of the order of [(0.005×0.3′)∧11)] ≈ 2×10^−31^. Thus analysis of singleton mutations is an excellent indication of new mutations and does not reflect sequencing errors (see also Results).

These SNPs were used to estimate the mutational profile of *S. aureus* in ex-operonic intergenic sites. Singletons are SNPs which are seen only once throughout all sequenced isolates. Such SNPs are more likely to represent recent mutational events which selection has not yet had time to act upon, and thus only singletons were considered in order to orientate the direction of changes and minimize the possibility of selection or multiple hits. As all other lineages (aside from that with the singleton) have the same nucleotide at the given location, the assignment of the ancestral state is unambiguous.

As we find the sample size of 13 SNPs falling within intra-operonic intergenic regions too small to calculate the relative mutation matrix for intra-operonic sites, we considered SNPs in ex-operonic intergenic sites only. On both strands singletons were isolated in intergenic sites outside operons and relative rates of mutation were calculated for the leading and lagging strands separately. Nucleotide frequencies at mutational compositional equilibrium were derived from the relative mutation rates by considering that at compositional equilibrium, the loss of any given nucleotide must equal the net gain of that nucleotide at other sites:
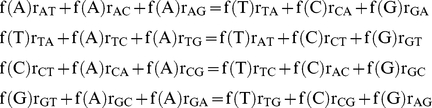
where f(i) is the frequency of site i and r_ij_ is the rate of change from i to j per site i as measured in the extant sequence. The above equilibrium equations were solved simultaneously using Maxima 5.21.1 [44] to yield equilibrium nucleotide frequencies. These equilibrium frequencies were used to calculate the AT skew in ex-operonic intergenic sites expected to result purely from replicational mutation at compositional equilibrium.

Similar mutational equilibrium analyses were performed on polymorphism data from *B. anthracis* and *S. typhi*. Intergenic singleton SNPs were extracted from alignments of 18 fully and partially sequenced *B. anthracis* strains [23] and from the intra-haplotype or haplotype-specific age groups for *S. typhi* SNP data [45]. For both organisms, only intergenic regions under 500 bp were considered and singletons were only called when sequence data was available for all strains and the SNP at least 60 bp away from a gene. Observed intergenic nucleotide content and AT skew were calculated using NCBI RefSeqs NC_003997 and NC_003198.

To obtain an approximate measure of the robustness the sign of the equilibrium AT skew indicated by the singleton SNP populations, the intergenic (ex-operonic in the case of *S. aureus*) SNPs were bootstrapped. For each species, the intergenic SNPs used to compute the mutational equilibrium were resampled with replacement 1000 times, and the equilibrium state recalculated as above after each resampling, to yield 95% bootstrap intervals for the equilibrium AT skew estimate.

### Randomizations

Selection against stop codons within asymmetrically distributed genes could necessarily impose some amount of AT skew as T might be underrepresented relative to A within first codon sites. We wished to measure the AT skew which results in *S. aureus* from selection on gene position alone while preventing any selection on amino acid content, which might further increase or decrease the amount of T relative to A within genes in *S. aureus*, from biasing this measurement. Randomized coding sequences provide a means of estimating the AT skew that would result from the biased gene orientation seen in *S. aureus* even under a complete lack of selection for amino-acid usage. As both GC content and replication-associated mutational biases can modulate the amino acid content of proteins [46], [47], the baseline nucleotide frequencies of the leading and lagging strands of the TW20 chromosome could favor the presence of certain codons while disfavoring others. A null was devised in which nucleotides were sampled in proportion to their frequency in intergenic regions, which should be neutral or weakly selected, thus controlling for the baseline nucleotide content of the genome as well as any mutational effect on skew. 10,000 protein-coding sequences containing the same number of amino acid-encoding codons as in the leading and lagging strands of the TW20 chromosome were simulated using codons derived from the intergenic nucleotide frequencies in the relevant strand of the TW20 chromosome. Stop and start codons were excluded from randomized sequences. Amino acids with six codons were considered as two separate amino acids—a 4-block and a 2-block—since the frequency of individual nucleotides could differentially influence the usage of these two codon blocks. The resulting AT skew in randomized chromosomes was calculated in first and second sites as (A−T)/(A+T) with respect to the sense direction.

Selective patterns of amino acid usage may, depending on the nucleotide frequencies of the codons involved, also shape AT skew. Determination of whether individual amino acids are over- or under-used in relation to the above null is reflected in the Z score for each amino acid (aa):
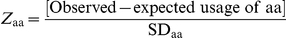
where the expected usage is the mean usage of that amino acid amongst the 10000 simulated coding regions, the observed usage is that seen in the TW20 chromosome and the standard deviation is that observed through the randomizations. This normalizes for variance seen due to amino acids occupying differing amounts of codon space, controls for the effect that genomic GC content may have on individual codon usage, and allows for comparison of over- or under-usage across different amino acids.

### Gene strandedness and genomic AT skew across Firmicutes

If gene strand bias is responsible for positive AT skews not just within *S. aureus* but across the Firmicutes, we expect a positive association between gene strandedness and genomic AT skews across the phylum. A simple test for correlation between these two quantities across a wide sampling of Firmicute species might, however, falsely infer a relationship between the two due to over-representation of sequence information in closely related genomes. We therefore investigated the relationship between strand bias and genomic AT skew using phylogenetically independent contrasts. Differences in strand bias and leading genomic AT skew were calculated for phylogenetically independent pairs of terminal node species in a phylogeny of Firmicutes [48] (Table S3) with the expectation that if strand bias does dictate the extent of positive AT skew, an increase in strand bias between species should also result in an increase in AT skew. *Gespi* values, calculated according to de Carvalho & Ferreira 2007 [49], were used as indicators of the degree of strand bias among these species, with a higher *gespi* indicating a greater degree of strandedness. As a counterpoint to the Firmicutes, similar analyses were performed on phylogenies of the Gram negative Alpha-proteobacteria [32], Delta-proteobacteria [33], Epsilon-proteobacteria [34], Gamma-proteobacteria [35], and a phylogeny of the Gram positive Actinobacteria [36] (Tables S4, S5, S6, S7, S8).

## Supporting Information

Figure S1 *B. anthracis* and *S. typhi* both show fluctuations in AT skew in intergenic regions at gene boundaries. AT skew at each position was calculated from the nucleotide content measured across all intergenic regions at that position relative to the gene start or end as appropriate. All intergenic regions were considered in the direction of transcription of the relevant gene.
(DOC)

Figure S2 Z versus amino acid cost using alternative cost measure *A_glucose._*. A positive *Z* represents over-usage, a negative *Z* under-usage. Correlation between Z and amino acid cost, Spearman's rho: leading strand, -0.376, one-sided P = 0.038, lagging strand rho, -0.399, P = 0.031.
(DOC)

Figure S3 Z versus amino acid cost using alternative cost measure *R_glucose._*. A positive *Z* represents over-usage, a negative *Z* under-usage. Correlation between Z and amino acid cost, Spearman's rho: leading strand, one-sided P = 0.553, lagging strand, P = 0.553.
(DOC)

Figure S4 Z versus amino acid cost using the alternative cost measure of Craig and Weber energy. A positive *Z* represents over-usage, a negative *Z* under-usage. Correlation between Z and amino acid cost, Spearman's rho: leading strand, -0.578, one-sided P = 0.002, lagging strand rho, -0.566, P = 0.002.
(DOC)

Figure S5 Z versus amino acid cost using the alternative cost measure of Craig and Weber steps. A positive *Z* represents over-usage, a negative *Z* under-usage. Correlation between Z and amino acid cost, Spearman's rho, leading strand, -0.450, P = 0.016, lagging strand rho, -0.484, P = 0.009.
(DOC)

Figure S6 Z versus amino acid cost using the alternative cost measure of Wagner fermentative costs. A positive *Z* represents over-usage, a negative *Z* under-usage. Correlation between Z and amino acid cost, Spearman's rho, leading strand, -0.373, P = 0.040, lagging strand rho, -0.411, P = 0.026.
(DOC)

Figure S7 Z versus amino acid cost using the alternative cost measure of Wagner respiratory costs. A positive *Z* represents over-usage, a negative *Z* under-usage. Correlation between Z and amino acid cost, Spearman's rho, leading strand, -0.584, P = 0.002, lagging strand rho, -0.548, P = 0.003.
(DOC)

Figure S8 Z versus amino acid cost using alternative cost measure of molecular weight. A positive *Z* represents over-usage, a negative *Z* under-usage. Correlation between Z and amino acid cost, Spearman's rho, leading strand, P = 0.119, lagging strand, P = 0.079.
(DOC)

Figure S9 The observed cost of amino acids encoded in GC-poor genomes is lower than expected, suggesting more efficient cost selection in AT-rich bacteria. Coding sequences were simulated taking into account the GC contents of individual codon positions in the given strain and stop codon avoidance, with the number and length of simulated sequences based on observed values, assuming no GC or AT skew. (A) Box plot for 105 genomes, light blue: median simulated, dark blue: median observed Akashi and Gojobori [29] biosynthetic cost of amino acids encoded in genes, the lower and upper quartiles are shown in gray. (B) The factor by which the mean observed amino acid cost values are lower than expected correlates with genomic GC content. Orange: Firmicutes, Spearman's rho = −0.840, P<2.2×10^−16^, black: non-Firmicutes, Spearman's rho = −0.768, P<2.2×10^−16^, Firmicutes and non-Firmicutes together: Spearman's rho = −0.871, P-value <2.2×10^−16^.
(DOC)

Figure S10 AT skews correlate with the intensity of selection against costly amino acids. The ordinate shows the mean of AT skews calculated for individual protein coding genes in their sense direction in a given genome, for which the cost ratio was calculated as in Figure S9. Orange: Firmicutes, black: non-Firmicutes; see Table S2 for statistical data.
(DOC)

Figure S11 Outlier AT skew values are not responsible for the positive AT skews seen in ex-operonic intergenic regions. For each AT content observed in such an intergenic region in the TW20 genome, 1000 randomized sequences were created by shuffling the total nucleotide content in ex-operonic intergenic sequences 1000 times, and each time the shuffled sequence was repartitioned into intergenic regions containing the same AT contents as in the observed genome. The 95% confidence interval (black points) was calculated from these simulated sequences to determine which observed ex-operonic intergenic AT skew values (green points) were outliers (green filled points falling outside the 95% confidence interval). The leading AT skew in ex-operonic intergenic sequences with outliers removed (0.0257) is very similar to the same calculation inclusive of outliers (0.0276).
(DOC)

Table S1 a. Relative mutation rates of nucleotide i to j per site i for intergenic sites were calculated from singleton SNPs for *B. anthracis* (gray rows) and *S. typhi* (white rows). All rates are shown with respect to the leading strand and derived from the following leading strand SNP counts, where XY indicates a change from nucleotide XY: *B. anthracis* SNPs, AG 9 GA 11 GC 1 CG 1 GT 4 TA 2 TC 20 TG 2 CA 3 AC 3 AT 5 CT 15. *S. typhi* SNPs, AG 6 GA 15 GC 0 CG 0 GT 3 TA 0 TC 4 TG 0 AC 0 CA 1 AT 0 CT 14. b. Current observed intergenic AT skew contrasted with SNP-derived intergenic equilibrium AT skews for *B. anthracis* and *S. typhi*. All skews are given with respect to the leading strand. 95% bootstrap intervals are shown in parentheses. That *B. anthracis* does not display a consistently negative bootstrap interval is a consequence of at least two factors. Firstly, the sample size (76 SNPs) used to derived the mutational equilibrium is small compared to that used for *S. aureus* (140 SNPs). Secondly, the alignments used to derive the *B. anthracis* SNPs come from several independent sequencing efforts and we are unable to verify the sequence qualities. As for *S. typhi*, the even smaller sample size of 43 SNPs leaves many mutational categories unrepresented and leads to inflated bootstrap intervals.
(DOC)

Table S2 Spearman rank correlations between *Z* and amino acid cost using alternative cost measures.
(DOC)

Table S3 Terminal node comparisons taken from a phylogeny of Firmicutes [48] used to calculate the difference in *gespi* and leading strand genomic AT skew (where more than one species is listed in a field, the average of those genomes was taken).
(DOC)

Table S4 Terminal node comparisons taken from a phylogeny of Actinobacteria [36] used to calculate the difference in *gespi* and leading strand genomic AT skew.
(DOC)

Table S5 Terminal node comparisons taken from a phylogeny of Alpha-proteobacteria [32] used to calculate the difference in *gespi* and leading strand genomic AT skew.
(DOC)

Table S6 Terminal node comparisons taken from a phylogeny of Delta-proteobacteria [33] used to calculate the difference in *gespi* and leading strand genomic AT skew.
(DOC)

Table S7 Terminal node comparisons taken from a phylogeny of Epsilon-proteobacteria [34] used to calculate the difference in *gespi* and leading strand genomic AT skew.
(DOC)

Table S8 Terminal node comparisons taken from a phylogeny of Gamma-proteobacteria [35] used to calculate the difference in *gespi* and leading strand genomic AT skew (where more than one species is listed in a field, the average of those genomes was taken).
(DOC)

Table S9 Relationships between mean CDS AT skews and cost ratios calculated for eight different amino acid cost measures. C&W: Craig and Weber, Wagner ferm.: Wagner fermentative costs, Wagner resp.: Wagner respiratory costs. Amino acid cost ratios were calculated as in Figure S9.
(DOC)

Table S10 Coverage data for the 140 singleton ex-operonic intergenic SNPs used in this analysis. 126/140 (90%) of the SNPs were consistent called in all mapped reads (marked with a *). The minimum of consistent mapped reads was 12 (in four SNPs), and there was one SNP with 12 consistent reads and one inconsistent. For the 14 SNPs with inconsistent reads, all showed only a single inconsistent read bar one (which had 2 inconsistent reads). Given a sequence error rate of 0.5%, a high level of coverage and high consistency between reads, the probability that any of these SNPs are errors is negligible.
(DOC)
