# Supplementary figures and images for: Atypical AT Skew in Firmicute Genomes Results from Selection and Not from Mutation
Source: PLoS Genet. 2011 Sep 15;7(9):e1002283. doi: 10.1371/journal.pgen.1002283 (PMC3174206; doi:10.1371/journal.pgen.1002283)

**
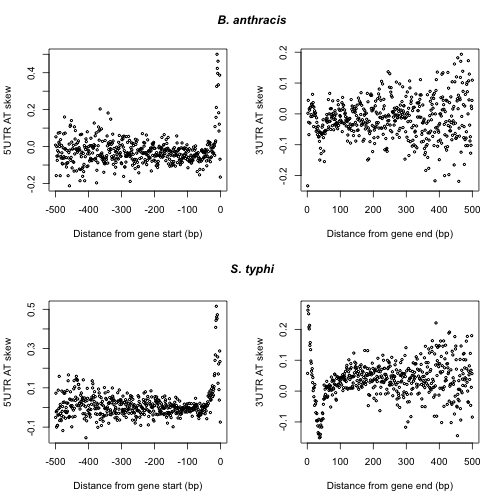
**

Supplement: Figure S1 — B. anthracis and S. typhi both show fluctuations in AT skew in intergenic regions at gene boundaries. AT skew at each position was calculated from the nucleotide content measured across all intergenic regions at that position relative to the gene start or end as appropriate. All intergenic regions were considered in the direction of transcription of the relevant gene. (DOC) [file pgen.1002283.s001.doc]

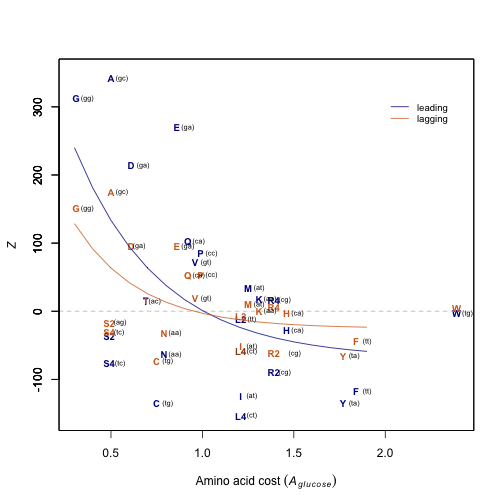

Supplement: Figure S2 — Z versus amino acid cost using alternative cost measure Aglucose.. A positive Z represents over-usage, a negative Z under-usage. Correlation between Z and amino acid cost, Spearman's rho: leading strand, -0.376, one-sided P = 0.038, lagging strand rho, -0.399, P = 0.031. (DOC) [file pgen.1002283.s002.doc]

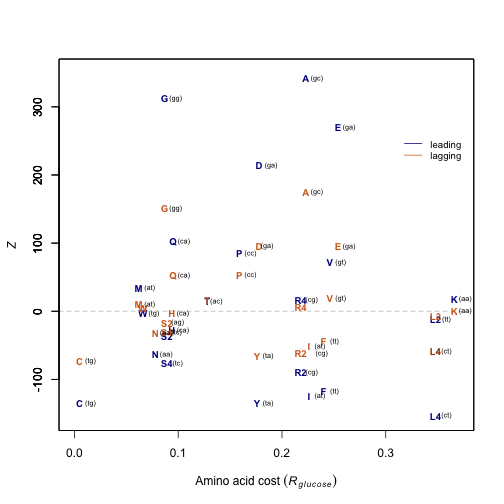

Supplement: Figure S3 — Z versus amino acid cost using alternative cost measure Rglucose.. A positive Z represents over-usage, a negative Z under-usage. Correlation between Z and amino acid cost, Spearman's rho: leading strand, one-sided P = 0.553, lagging strand, P = 0.553. (DOC) [file pgen.1002283.s003.doc]

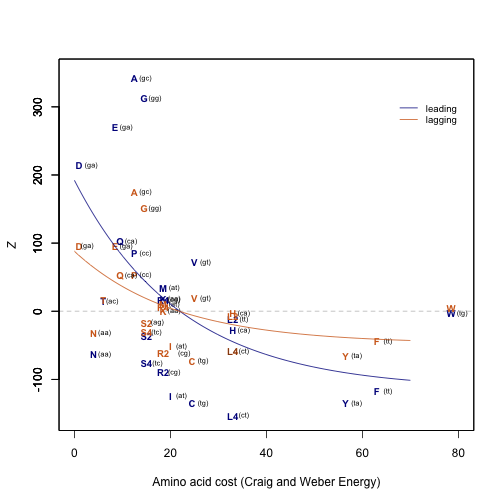

Supplement: Figure S4 — Z versus amino acid cost using the alternative cost measure of Craig and Weber energy. A positive Z represents over-usage, a negative Z under-usage. Correlation between Z and amino acid cost, Spearman's rho: leading strand, -0.578, one-sided P = 0.002, lagging strand rho, -0.566, P = 0.002. (DOC) [file pgen.1002283.s004.doc]

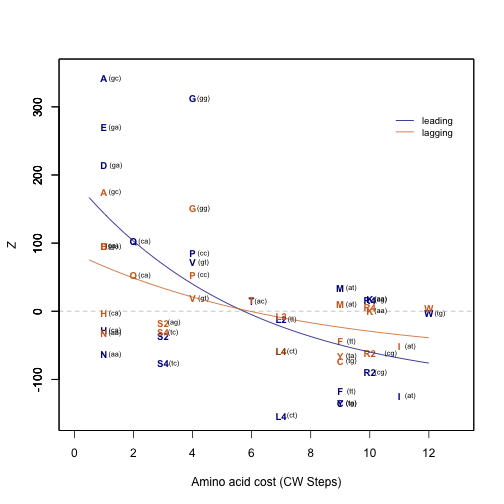

Supplement: Figure S5 — Z versus amino acid cost using the alternative cost measure of Craig and Weber steps. A positive Z represents over-usage, a negative Z under-usage. Correlation between Z and amino acid cost, Spearman's rho, leading strand, -0.450, P = 0.016, lagging strand rho, -0.484, P = 0.009. (DOC) [file pgen.1002283.s005.doc]

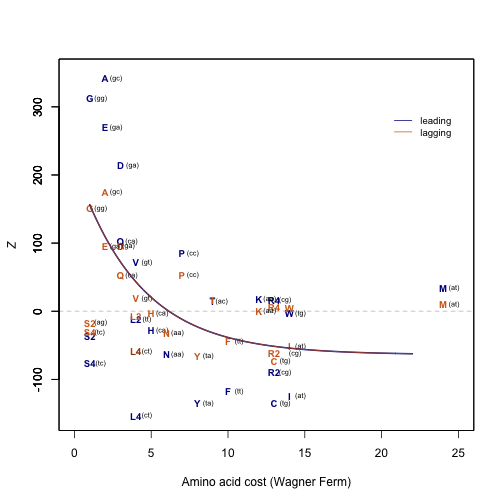

Supplement: Figure S6 — Z versus amino acid cost using the alternative cost measure of Wagner fermentative costs. A positive Z represents over-usage, a negative Z under-usage. Correlation between Z and amino acid cost, Spearman's rho, leading strand, -0.373, P = 0.040, lagging strand rho, -0.411, P = 0.026. (DOC) [file pgen.1002283.s006.doc]

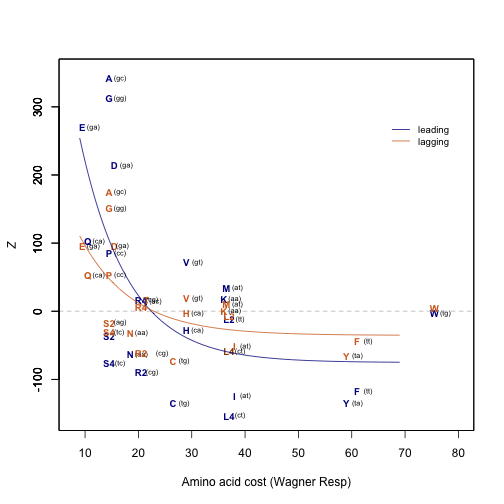

Supplement: Figure S7 — Z versus amino acid cost using the alternative cost measure of Wagner respiratory costs. A positive Z represents over-usage, a negative Z under-usage. Correlation between Z and amino acid cost, Spearman's rho, leading strand, -0.584, P = 0.002, lagging strand rho, -0.548, P = 0.003. (DOC) [file pgen.1002283.s007.doc]

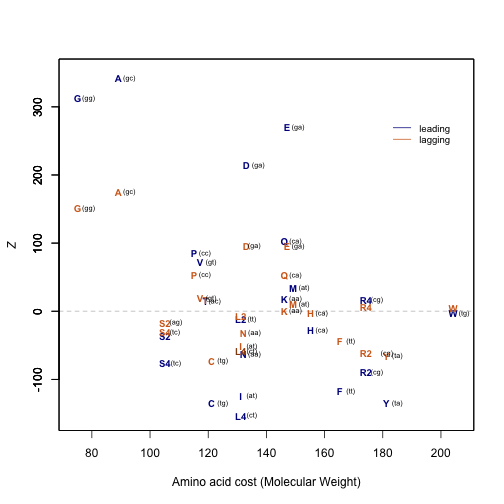

Supplement: Figure S8 — Z versus amino acid cost using alternative cost measure of molecular weight. A positive Z represents over-usage, a negative Z under-usage. Correlation between Z and amino acid cost, Spearman's rho, leading strand, P = 0.119, lagging strand, P = 0.079. (DOC) [file pgen.1002283.s008.doc]

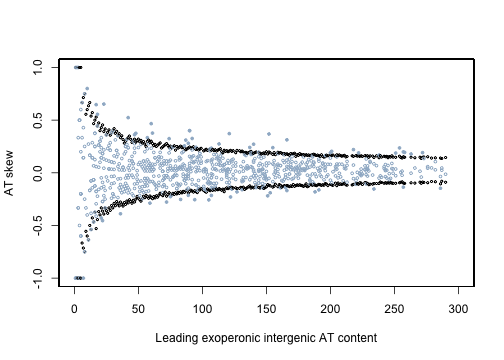

Supplement: Figure S11 — Outlier AT skew values are not responsible for the positive AT skews seen in ex-operonic intergenic regions. For each AT content observed in such an intergenic region in the TW20 genome, 1000 randomized sequences were created by shuffling the total nucleotide content in ex-operonic intergenic sequences 1000 times, and each time the shuffled sequence was repartitioned into intergenic regions containing the same AT contents as in the observed genome. The 95% confidence interval (black points) was calculated from these simulated sequences to determine which observed ex-operonic intergenic AT skew values (green points) were outliers (green filled points falling outside the 95% confidence interval). The leading AT skew in ex-operonic intergenic sequences with outliers removed (0.0257) is very similar to the same calculation inclusive of outliers (0.0276). (DOC) [file pgen.1002283.s011.doc]
