# Supplementary material for: Atypical AT Skew in Firmicute Genomes Results from Selection and Not from Mutation
Source: PLoS Genet. 2011 Sep 15;7(9):e1002283. doi: 10.1371/journal.pgen.1002283 (PMC3174206; doi:10.1371/journal.pgen.1002283)
Supplement: Table S1 — a. Relative mutation rates of nucleotide i to j per site i for intergenic sites were calculated from singleton SNPs for B. anthracis (gray rows) and S. typhi (white rows). All rates are shown with respect to the leading strand and derived from the following leading strand SNP counts, where XY indicates a change from nucleotide XY: B. anthracis SNPs, AG 9 GA 11 GC 1 CG 1 GT 4 TA 2 TC 20 TG 2 CA 3 AC 3 AT 5 CT 15. S. typhi SNPs, AG 6 GA 15 GC 0 CG 0 GT 3 TA 0 TC 4 TG 0 AC 0 CA 1 AT 0 CT 14. b. Current observed intergenic AT skew contrasted with SNP-derived intergenic equilibrium AT skews for B. anthracis and S. typhi. All skews are given with respect to the leading strand. 95% bootstrap intervals are shown in parentheses. That B. anthracis does not display a consistently negative bootstrap interval is a consequence of at least two factors. Firstly, the sample size (76 SNPs) used to derived the mutational equilibrium is small compared to that used for S. aureus (140 SNPs). Secondly, the alignments used to derive the B. anthracis SNPs come from several independent sequencing efforts and we are unable to verify the sequence qualities. As for S. typhi, the even smaller sample size of 43 SNPs leaves many mutational categories unrepresented and leads to inflated bootstrap intervals. (DOC) [file pgen.1002283.s012.doc]

**a.**

|  | from  A | T | C | G | Equilibrium frequency |
| --- | --- | --- | --- | --- | --- |
| to  A | - | 1.1120007e-5 | 4.2321474e-5 | 0.0001115653 | .29778 |
|  | - | 0.0 | 9.1101232e-6 | 0.0001347104 | .16819 |
| T | 2.6191998e-5 | - | 0.0002116074 | 4.0569186e-5 | .38923 |
|  | 0.0 | - | 0.0001275417 | 2.6942074540 | .63886 |
| C | 1.5715199e-5 | 0.0001112001 | - | 1.0142296e-5 | .18383 |
|  | 0.0 | 3.0842779e-5 | - | 0.0 | .14419 |
| G | 4.7145596e-5 | 1.1120007e-5 | 1.4107158e-5 | - | .12917 |
|  | 4.6861820e-5 | 0.0 | 0.0 | - | .04876 |

**b.**

|  | ***B. anthracis*** | ***S. typhi*** |
| --- | --- | --- |
| **Observed intergenic AT skew** | +0.0298 | -0.0064 |
| **Equilibrium intergenic AT skew** | -0.1331 (-0.6002, +0.3609) | -0.0583 (-1, 1) |
