# Supplementary material for: Atypical AT Skew in Firmicute Genomes Results from Selection and Not from Mutation
Source: PLoS Genet. 2011 Sep 15;7(9):e1002283. doi: 10.1371/journal.pgen.1002283 (PMC3174206; doi:10.1371/journal.pgen.1002283)
Supplement: Table S2 — Spearman rank correlations between Z and amino acid cost using alternative cost measures. (DOC) [file pgen.1002283.s013.doc]

|  | **Leading** | | **Lagging** | |
| --- | --- | --- | --- | --- |
| **Cost measure** | **Spearman rho** | **P (one-sided)** | **Spearman rho** | **P (one-sided)** |
| *A_glucose_* | -0.376 | 0.038 | -0.399 | 0.031 |
| *R_glucose_* | - | 0.553 | - | 0.553 |
| Craig and Weber energy | -0.578 | 0.002 | -0.566 | 0.002 |
| Craig and Weber steps | -0.450 | 0.016 | -0.484 | 0.009 |
| Wagner fermentation | -0.373 | 0.040 | -0.411 | 0.026 |
| Wagner respiration | -0.584 | 0.002 | -0.548 | 0.003 |
| Molecular weight | - | 0.119 | - | 0.079 |
