# Supplementary material for: Atypical AT Skew in Firmicute Genomes Results from Selection and Not from Mutation
Source: PLoS Genet. 2011 Sep 15;7(9):e1002283. doi: 10.1371/journal.pgen.1002283 (PMC3174206; doi:10.1371/journal.pgen.1002283)
Supplement: Table S3 — Terminal node comparisons taken from a phylogeny of Firmicutes [48] used to calculate the difference in gespi and leading strand genomic AT skew (where more than one species is listed in a field, the average of those genomes was taken). (DOC) [file pgen.1002283.s014.doc]

| **Firmicutes** | |
| --- | --- |
| **Terminal node 1** | **Terminal node 2** |
| NC_003454 *Fusobacterium nucleatum* | NC_003212 *Listeria innocua* |
| NC_010503 *Ureaplasma parvum* | NC_004432 *Mycoplasma penetrans* |
| NC_007332 *Mycoplasma hyopneumoniae* | NC_002771 *Mycoplasma pulmonis* |
| NC_007633 *Mycoplasma capricolum* | NC_005364 *Mycoplasma mycoides* |
| NC_009012 *Clostridium thermocellum* | NC_003869 *Thermoanaerobacter tengcongensis* |
| NC_000964 *Bacillus subtilis* | NC_005945 *Bacillus anthracis* |
| NC_014103 *Bacillus megaterium* | NC_002570 *Bacillus halodurans* |
| NC_004557 *Clostridium tetani* | NC_003366 *Clostridium perfringens* |
| NC_002662 *Lactococcus lactis*, NC_013656 *Lactococcus lactis* | AE014133 *Streptococcus mutans* |
| CP000419 *Streptococcus thermophilus*, NC_006448 *Streptococcus thermophilus*, NC_006449 *Streptococcus thermophilus* | FM211187 *Streptococcus pneumoniae* |
| NC_004116 *Streptococcus agalactiae*, NC_004368 *Streptococcus agalactiae*, NC_007432 *Streptococcus agalactiae* | NC_004070 *Streptococcus pyogenes*, NC_004606 *Streptococcus pyogenes*, NC_007297 *Streptococcus pyogenes*, NC_008021 *Streptococcus pyogenes*, NC_008022 *Streptococcus pyogenes*, NC_008023 *Streptococcus pyogenes*, NC_008024 *Streptococcus pyogenes*, NC_009332 *Streptococcus pyogenes*, NC_011375 *Streptococcus pyogenes* |
| AE015929 *Staphylococcus epidermidis*, NC_007168 *Staphylococcus haemolyticus*,  NC_007350 *Staphylococcus saprophyticus*,  NC_007793 *Staphylococcus aureus*, NC_012121 *Staphylococcus carnosus*, NC_014925 *Staphylococcus pseudintermedius* | NC_003454 *Fusobacterium nucleatum* |
| NC_002973 *Listeria monocytogenes*, NC_003210 *Listeria monocytogenes*, NC_003212 *Listeria innocua*,  NC_011660 *Listeria monocytogenes*, NC_012488 *Listeria monocytogenes* | NC_004668 *Enterococcus faecalis* |
| NC_008530 *Lactobacillus gasseri*, NC_014554 *Lactobacillus plantarum* | CP000414 *Leuconostoc mesenteroides* |
