# Supplementary material for: Atypical AT Skew in Firmicute Genomes Results from Selection and Not from Mutation
Source: PLoS Genet. 2011 Sep 15;7(9):e1002283. doi: 10.1371/journal.pgen.1002283 (PMC3174206; doi:10.1371/journal.pgen.1002283)
Supplement: Table S4 — Terminal node comparisons taken from a phylogeny of Actinobacteria [36] used to calculate the difference in gespi and leading strand genomic AT skew. (DOC) [file pgen.1002283.s015.doc]

| **Actinobacteria** | |
| --- | --- |
| **Terminal node 1** | **Terminal node 2** |
| NC_013929 *Streptomyces scabiei* | NC_013131 *Catenulispora acidiphila* |
| NC_011886 *Arthrobacter chlorophenolicus* | NC_010168 *Renibacterium salmoninarum* |
| NC_014643 *Rothia dentocariosa* | NC_010617 *Kocuria rhizophila* |
| NC_010407 *Clavibacter michiganensis* | NC_006087 *Leifsonia xyli* |
| NC_013521 *Sanguibacter keddiei* | NC_013174 *Jonesia denitrificans* |
| NC_014218 *Arcanobacterium haemolyticum* | NC_012669 *Beutenbergia cavernae* |
| NC_013721 *Gardnerella vaginalis* | NC_014616 *Bifidobacterium bifidum* |
| NC_002755 *Mycobacterium tuberculosis* | NC_002935 *Corynebacterium diphtheriae* |
| NC_006361 *Nocardia farcinica* | NC_014168 *Segniliparus rotundus* |
| NC_013159 *Saccharomonospora viridis* | NC_009142 *Saccharopolyspora erythraea* |
| NC_014391 *Micromonospora_aurantiaca* | NC_009953 *Salinispora arenicola* |
| NC_014165 *Thermobispora bispora* | NC_013947 *Stackebrandtia nassauensis* |
| NC_008699 *Nocardioides sp.* | NC_013729 *Kribbella flavida* |
| NC_013595 *Streptosporangium roseum* | NC_013510 *Thermomonospora curvata* |
| NC_014210 *Nocardiopsis dassonvillei* | NC_007333 *Thermobifida fusca* |
| NC_013235 *Nakamurella multipartita* | NC_008578 *Acidothermus cellulolyticus* |
| NC_013204 *Eggerthella lenta* | NC_014363 *Olsenella uli* |
