# Supplementary material for: Atypical AT Skew in Firmicute Genomes Results from Selection and Not from Mutation
Source: PLoS Genet. 2011 Sep 15;7(9):e1002283. doi: 10.1371/journal.pgen.1002283 (PMC3174206; doi:10.1371/journal.pgen.1002283)
Supplement: Table S5 — Terminal node comparisons taken from a phylogeny of Alpha-proteobacteria [32] used to calculate the difference in gespi and leading strand genomic AT skew. (DOC) [file pgen.1002283.s016.doc]

| **Alpha-proteobacteria** | |
| --- | --- |
| **Terminal node 1** | **Terminal node 2** |
| NC_004310 *Brucella suis* | NC_008783 *Bartonella bacilliformis* |
| NC_002678 *Mesorhizobium loti* | NC_003047 *Sinorhizobium meliloti* |
| NC_007958 *Rhodopseudomonas palustris* | NC_007406 *Nitrobacter winogradskyi* |
| NC_004463 *Bradyrhizobium japonicum* | NC_009720 *Xanthobacter autotrophicus* |
| NC_003911 *Ruegeria pomeroyi* | NC_007802 *Jannaschia sp.* |
| NC_009428 *Rhodobacter sphaeroides* | NC_008686 *Paracoccus denitrificans* |
| NC_002696 *Caulobacter crescentus* | NC_008347 *Maricaulis maris* |
| NC_007722 *Erythrobacter litoralis* | NC_007794 *Novosphingobium aromaticivorans* |
| NC_008048 *Sphingopyxis alaskensis* | NC_006526 *Zymomonas mobilis* |
| NC_006677 *Gluconobacter oxydans* | NC_009484 *Acidiphilium cryptum* |
| NC_007626 *Magnetospirillum magneticum* | NC_007643 *Rhodospirillum rubrum* |
| NC_006832 *Ehrlichia ruminantium* | NC_004842 *Anaplasma marginale* |
| NC_007797 *Anaplasma phagocytophilum* | NC_002978 *Wolbachia endosymbiont* |
| NC_007940 *Rickettsia bellii* | NC_007205 *Candidatus Pelagibacter* |
