# Supplementary material for: Atypical AT Skew in Firmicute Genomes Results from Selection and Not from Mutation
Source: PLoS Genet. 2011 Sep 15;7(9):e1002283. doi: 10.1371/journal.pgen.1002283 (PMC3174206; doi:10.1371/journal.pgen.1002283)
Supplement: Table S6 — Terminal node comparisons taken from a phylogeny of Delta-proteobacteria [33] used to calculate the difference in gespi and leading strand genomic AT skew. (DOC) [file pgen.1002283.s017.doc]

| **Delta-proteobacteria** | |
| --- | --- |
| **Terminal node 1** | **Terminal node 2** |
| NC_011768 *Desulfatibacillum alkenivorans* | NC_006138 *Desulfotalea psychrophila* |
| NC_014972 *Desulfobulbus propionicus* | NC_014216 *Desulfurivibrio alkaliphilus* |
| NC_008554 *Syntrophobacter fumaroxidans* | NC_015388 *Desulfobacca acetoxidans* |
| NC_014365 *Desulfarculus baarsii* | NC_005363 *Bdellovibrio bacteriovorus* |
| NC_007498 *Pelobacter carbinolicus* | NC_002939 *Geobacter sulfurreducens* |
| NC_008095 *Myxococcus xanthus* | NC_011891 *Anaeromyxobacter dehalogenans* |
| NC_013440 *Haliangium ochraceum* | NC_010162 *Sorangium cellulosum* |
| NC_007519 *Desulfovibrio desulfuricans* | NC_008011 *Lawsonia intracellularis* |
| NC_013173 *Desulfomicrobium baculatum* | NC_013223 *Desulfohalobium retbaense* |
