# Supplementary material for: Atypical AT Skew in Firmicute Genomes Results from Selection and Not from Mutation
Source: PLoS Genet. 2011 Sep 15;7(9):e1002283. doi: 10.1371/journal.pgen.1002283 (PMC3174206; doi:10.1371/journal.pgen.1002283)
Supplement: Table S7 — Terminal node comparisons taken from a phylogeny of Epsilon-proteobacteria [34] used to calculate the difference in gespi and leading strand genomic AT skew. (DOC) [file pgen.1002283.s018.doc]

| **Epsilon-proteobacteria** | |
| --- | --- |
| **Terminal node 1** | **Terminal node 2** |
| NC_012115 *Nautilia profundicola* | NC_000921 *Helicobacter pylori* |
| NC_014935 *Nitratifractor salsuginis* | NC_009663 *Sulfurovum sp.* |
| NC_013512 *Sulfurospirillum deleyianum* | NC_009839 *Campylobacter jejuni* |
| NC_014506 *Sulfurimonas autotrophica* | NC_007575 *Sulfurimonas denitrificans* |
