# Supplementary material for: Atypical AT Skew in Firmicute Genomes Results from Selection and Not from Mutation
Source: PLoS Genet. 2011 Sep 15;7(9):e1002283. doi: 10.1371/journal.pgen.1002283 (PMC3174206; doi:10.1371/journal.pgen.1002283)
Supplement: Table S8 — Terminal node comparisons taken from a phylogeny of Gamma-proteobacteria [35] used to calculate the difference in gespi and leading strand genomic AT skew (where more than one species is listed in a field, the average of those genomes was taken). (DOC) [file pgen.1002283.s019.doc]

| **Gamma-proteobacteria** | |
| --- | --- |
| **Terminal node 1** | **Terminal node 2** |
| NC_003143 *Yersinia pestis*,  NC_004088 *Yersinia pestis* | NC_005126 *Photorhabdus luminescens* |
| NC_004547 *Pectobacterium atrosepticum* | NC_007712 *Sodalis glossinidius* |
| NC_003197 *Salmonella enterica*, NC_003198 *Salmonella enterica* | NC_000913 *Escherichia coli*, NC_010473 *Escherichia coli* |
| NC_002528 *Buchnera aphidicola* | NC_008513 *Buchnera aphidicola* |
| NC_002663 *Pasteurella multocida* | NC_006840 *Vibrio fischeri* |
| NC_008570 *Aeromonas hydrophila* | NC_008709 *Psychromonas ingrahamii* |
| NC_007481 *Pseudoalteromonas haloplanktis* | NC_008700 *Shewanella amazonensis* |
| NC_002947 *Pseudomonas putida*, NC_004129 *Pseudomonas fluorescens*, NC_004578 *Pseudomonas syringae*, NC_007492 *Pseudomonas fluorescens* | NC_008740 *Marinobacter aquaeolei* |
| NC_005966 *Acinetobacter sp*. | NC_008260 *Alcanivorax borkumensis* |
| NC_008610 *Candidatus Ruthia*, NC_009465 *Candidatus Vesicomyosocius* | NC_007520 *Thiomicrospira crunogena* |
| NC_009446 *Dichelobacter nodosus* | NC_006570 *Francisella tularensis* |
| NC_002971 *Coxiella burnetii* | NC_002942 *Legionella pneumophila* |
| NC_008340 *Alkalilimnicola ehrlichii* | NC_003902 *Xanthomonas campestris* |
