# Supplementary material for: Atypical AT Skew in Firmicute Genomes Results from Selection and Not from Mutation
Source: PLoS Genet. 2011 Sep 15;7(9):e1002283. doi: 10.1371/journal.pgen.1002283 (PMC3174206; doi:10.1371/journal.pgen.1002283)
Supplement: Table S9 — Relationships between mean CDS AT skews and cost ratios calculated for eight different amino acid cost measures. C&W: Craig and Weber, Wagner ferm.: Wagner fermentative costs, Wagner resp.: Wagner respiratory costs. Amino acid cost ratios were calculated as in Figure S9. (DOC) [file pgen.1002283.s020.doc]

| **Cost measure** | **Group** | **Spearman’s rho** | **P-value** |
| --- | --- | --- | --- |
| **Akashi & Gojobori** | All | 0.737 | <2.2x10^-16^ |
|  | Firmicutes | 0.631 | 1.0x10^-6^ |
|  | Non-Firmicutes | 0.581 | 7.6x10^-6^ |
| **A_glucose_** | All | 0.606 | <2.2x10^-16^ |
|  | Firmicutes | 0.561 | 2.1x10^-5^ |
|  | Non-Firmicutes | 0.438 | 1.2x10^-3^ |
| **R_glucose_** | All | 0.312 | 1.3x10^-3^ |
|  | Firmicutes | 0.262 | 0.0609 |
|  | Non-Firmicutes | 0.229 | 0.0984 |
| **Craig & Weber** | All | 0.836 | <2.2x10^-16^ |
|  | Firmicutes | 0.713 | 1.3x10^-8^ |
|  | Non-Firmicutes | 0.767 | <2.2x10^-16^ |
|  | All | 0.599 | <2.2x10^-16^ |
| **C&W steps** | Firmicutes | 0.497 | 2.2x10^-4^ |
|  | Non-Firmicutes | 0.423 | 1.7x10^-3^ |
|  | All | -0.024 | 0.811 |
| **Wagner ferm.** | Firmicutes | 0.180 | 0.201 |
|  | Non-Firmicutes | -0.316 | 0.0216 |
|  | All | 0.809 | <2.2x10^-16^ |
| **Wagner resp.** | Firmicutes | 0.698 | 3.7x10^-8^ |
|  | Non-Firmicutes | 0.683 | 6.2x10^-8^ |
|  | All | 0.263 | 7.0x10^-3^ |
| **Molecular weight** | Firmicutes | 0.473 | 4.7x10^-4^ |
|  | Non-Firmicutes | 0.014 | 0.922 |
