# Supplementary material for: Atypical AT Skew in Firmicute Genomes Results from Selection and Not from Mutation
Source: PLoS Genet. 2011 Sep 15;7(9):e1002283. doi: 10.1371/journal.pgen.1002283 (PMC3174206; doi:10.1371/journal.pgen.1002283)
Supplement: Table S10 — Coverage data for the 140 singleton ex-operonic intergenic SNPs used in this analysis. 126/140 (90%) of the SNPs were consistent called in all mapped reads (marked with a *). The minimum of consistent mapped reads was 12 (in four SNPs), and there was one SNP with 12 consistent reads and one inconsistent. For the 14 SNPs with inconsistent reads, all showed only a single inconsistent read bar one (which had 2 inconsistent reads). Given a sequence error rate of 0.5%, a high level of coverage and high consistency between reads, the probability that any of these SNPs are errors is negligible. (DOC) [file pgen.1002283.s021.doc]

| **Position in TW20** | **Strain** | **Ref Base** | **Snp Base** | **Coverage** | **A** | **C** | **G** | **T** |  |
| --- | --- | --- | --- | --- | --- | --- | --- | --- | --- |
| 3543 | 3HK | T | A | 32 | 32 | 0 | 0 | 0 | ***** |
| 119065 | GRE317 | T | C | 19 | 0 | 19 | 0 | 0 | ***** |
| 119070 | AGT9 | C | A | 14 | 14 | 0 | 0 | 0 | ***** |
| 136038 | URU34 | G | T | 14 | 0 | 0 | 0 | 14 | ***** |
| 169266 | URU110 | A | T | 24 | 0 | 0 | 0 | 24 | ***** |
| 169271 | ICP5011 | T | A | 15 | 15 | 0 | 0 | 0 | ***** |
| 195796 | ICP5014 | T | C | 25 | 0 | 24 | 0 | 1 |  |
| 216450 | HDG2 | C | A | 13 | 13 | 0 | 0 | 0 | ***** |
| 216571 | GRE108 | A | C | 19 | 1 | 18 | 0 | 0 |  |
| 225250 | HSA10 | G | T | 20 | 1 | 0 | 0 | 19 |  |
| 288184 | ICP5014 | C | T | 29 | 0 | 0 | 0 | 29 | ***** |
| 289381 | HGSA9 | G | A | 13 | 13 | 0 | 0 | 0 | ***** |
| 289522 | 2A8 | A | G | 34 | 0 | 0 | 34 | 0 | ***** |
| 312857 | R35 | G | T | 25 | 0 | 0 | 0 | 25 | ***** |
| 322488 | S7 | C | T | 46 | 0 | 0 | 0 | 46 | ***** |
| 323776 | GRE317 | C | T | 17 | 0 | 0 | 0 | 17 | ***** |
| 332966 | S71 | A | G | 19 | 0 | 0 | 19 | 0 | ***** |
| 348784 | URU34 | T | C | 14 | 0 | 13 | 1 | 0 |  |
| 419993 | URU110 | A | G | 29 | 0 | 0 | 29 | 0 | ***** |
| 422442 | ICP5014 | T | C | 34 | 0 | 33 | 1 | 0 |  |
| 432205 | S87 | T | A | 13 | 13 | 0 | 0 | 0 | ***** |
| 468070 | URU110 | A | T | 22 | 0 | 0 | 0 | 22 | ***** |
| 470627 | S38 | A | G | 18 | 0 | 0 | 18 | 0 | ***** |
| 475566 | LHH1 | G | T | 23 | 0 | 0 | 0 | 23 | ***** |
| 489841 | BK2491 | A | G | 26 | 0 | 0 | 26 | 0 | ***** |
| 490523 | GRE317 | A | G | 24 | 0 | 0 | 23 | 1 |  |
| 546668 | S38 | C | T | 22 | 0 | 1 | 0 | 21 |  |
| 556456 | BRA36 | A | G | 21 | 0 | 0 | 21 | 0 | ***** |
| 574221 | HU106 | G | T | 18 | 0 | 0 | 0 | 18 | ***** |
| 737692 | S7 | A | T | 23 | 0 | 0 | 0 | 23 | ***** |
| 751439 | HU109 | C | T | 14 | 0 | 0 | 0 | 14 | ***** |
| 768052 | TUR1 | T | A | 21 | 21 | 0 | 0 | 0 | ***** |
| 773184 | HGSA942 | C | T | 21 | 0 | 0 | 0 | 21 | ***** |
| 773355 | GRE4 | A | T | 14 | 0 | 0 | 0 | 14 | ***** |
| 805381 | URU110 | C | A | 25 | 25 | 0 | 0 | 0 | ***** |
| 824680 | TUR9 | G | A | 14 | 14 | 0 | 0 | 0 | ***** |
| 827513 | S38 | A | T | 21 | 2 | 0 | 0 | 19 |  |
| 839611 | HU106 | G | T | 18 | 0 | 0 | 0 | 18 | ***** |
| 894265 | S38 | T | C | 23 | 0 | 23 | 0 | 0 | ***** |
| 935616 | AGT1 | G | A | 21 | 21 | 0 | 0 | 0 | ***** |
| 989519 | CHI59 | A | G | 14 | 0 | 0 | 14 | 0 | ***** |
| 1015615 | URU110 | A | C | 30 | 0 | 30 | 0 | 0 | ***** |
| 1057598 | ICP5011 | A | C | 19 | 0 | 19 | 0 | 0 | ***** |
| 1073116 | TUR9 | G | T | 13 | 0 | 0 | 0 | 13 | ***** |
| 1090595 | S87 | A | G | 12 | 0 | 0 | 12 | 0 | ***** |
| 1100318 | GRE317 | C | T | 13 | 0 | 0 | 0 | 13 | ***** |
| 1157372 | S71 | G | A | 19 | 19 | 0 | 0 | 0 | ***** |
| 1172459 | S71 | A | T | 19 | 0 | 0 | 0 | 19 | ***** |
| 1177758 | URU34 | T | C | 13 | 0 | 13 | 0 | 0 | ***** |
| 1186364 | CHI59 | G | T | 13 | 0 | 0 | 0 | 13 | ***** |
| 1196452 | HUR18 | A | G | 13 | 0 | 0 | 13 | 0 | ***** |
| 1239593 | GRE4 | A | G | 15 | 0 | 0 | 15 | 0 | ***** |
| 1240667 | TUR9 | C | A | 15 | 15 | 0 | 0 | 0 | ***** |
| 1262367 | BRA36 | A | T | 14 | 0 | 0 | 0 | 14 | ***** |
| 1294998 | S130 | T | C | 13 | 0 | 13 | 0 | 0 | ***** |
| 1295177 | S25 | C | A | 16 | 15 | 1 | 0 | 0 |  |
| 1295178 | S25 | G | T | 16 | 0 | 0 | 1 | 15 |  |
| 1317261 | CHI59 | A | G | 25 | 0 | 0 | 25 | 0 | ***** |
| 1372095 | HU106 | T | C | 15 | 0 | 15 | 0 | 0 | ***** |
| 1378948 | HSA10 | A | G | 19 | 0 | 0 | 19 | 0 | ***** |
| 1384475 | TUR1 | T | C | 13 | 0 | 13 | 0 | 0 | ***** |
| 1426231 | CHL1 | G | T | 27 | 0 | 0 | 0 | 27 | ***** |
| 1429388 | S87 | T | C | 13 | 0 | 13 | 0 | 0 | ***** |
| 1454793 | BK2491 | A | G | 18 | 0 | 0 | 18 | 0 | ***** |
| 1459809 | S7 | A | G | 42 | 0 | 0 | 42 | 0 | ***** |
| 1521655 | URU110 | T | A | 26 | 26 | 0 | 0 | 0 | ***** |
| 1561833 | HU106 | A | T | 22 | 0 | 0 | 0 | 22 | ***** |
| 1561871 | TUR1 | C | A | 23 | 23 | 0 | 0 | 0 | ***** |
| 1561928 | ICP5011 | C | A | 15 | 15 | 0 | 0 | 0 | ***** |
| 1562019 | HSA10 | A | G | 18 | 0 | 0 | 18 | 0 | ***** |
| 1573814 | 3HK | C | A | 17 | 17 | 0 | 0 | 0 | ***** |
| 1622627 | S106 | A | T | 15 | 0 | 0 | 0 | 15 | ***** |
| 1662688 | HU106 | G | A | 13 | 13 | 0 | 0 | 0 | ***** |
| 1662901 | HSA10 | A | G | 19 | 0 | 0 | 19 | 0 | ***** |
| 1690182 | URU110 | C | A | 25 | 25 | 0 | 0 | 0 | ***** |
| 1758747 | URU110 | G | A | 25 | 24 | 0 | 1 | 0 |  |
| 1758893 | ICP5011 | T | C | 26 | 0 | 26 | 0 | 0 | ***** |
| 1783198 | 3HK | C | A | 17 | 17 | 0 | 0 | 0 | ***** |
| 1810035 | S71 | C | T | 21 | 0 | 0 | 0 | 21 | ***** |
| 1823209 | ICP5014 | T | C | 16 | 0 | 16 | 0 | 0 | ***** |
| 1840121 | URU110 | C | T | 18 | 0 | 0 | 0 | 18 | ***** |
| 1850946 | LHH1 | A | T | 26 | 0 | 0 | 0 | 26 | ***** |
| 1906982 | FFP103 | C | A | 16 | 16 | 0 | 0 | 0 | ***** |
| 1907023 | HUSA304 | T | C | 13 | 1 | 12 | 0 | 0 |  |
| 1927797 | R35 | T | C | 33 | 1 | 32 | 0 | 0 |  |
| 1930867 | GRE108 | A | G | 12 | 0 | 0 | 12 | 0 | ***** |
| 1941623 | HU109 | G | A | 20 | 20 | 0 | 0 | 0 | ***** |
| 1979048 | HUSA304 | G | A | 14 | 14 | 0 | 0 | 0 | ***** |
| 2034898 | URU110 | C | T | 15 | 0 | 0 | 0 | 15 | ***** |
| 2044745 | ICP5014 | A | G | 46 | 0 | 0 | 46 | 0 | ***** |
| 2068298 | S71 | A | G | 16 | 0 | 0 | 16 | 0 | ***** |
| 2108139 | CHI61 | G | A | 25 | 25 | 0 | 0 | 0 | ***** |
| 2170431 | CHI61 | C | G | 15 | 0 | 0 | 15 | 0 | ***** |
| 2180509 | S71 | T | C | 17 | 0 | 17 | 0 | 0 | ***** |
| 2312416 | HU106 | T | A | 14 | 14 | 0 | 0 | 0 | ***** |
| 2317121 | GRE108 | A | G | 14 | 0 | 0 | 14 | 0 | ***** |
| 2360959 | URU110 | T | C | 29 | 0 | 29 | 0 | 0 | ***** |
| 2369144 | AGT1 | G | A | 21 | 21 | 0 | 0 | 0 | ***** |
| 2397583 | URU110 | T | C | 17 | 0 | 17 | 0 | 0 | ***** |
| 2422692 | S102 | A | G | 16 | 0 | 0 | 16 | 0 | ***** |
| 2425126 | S7 | C | T | 27 | 0 | 0 | 0 | 27 | ***** |
| 2438390 | URU34 | C | A | 14 | 14 | 0 | 0 | 0 | ***** |
| 2453358 | URU110 | T | C | 30 | 1 | 29 | 0 | 0 |  |
| 2487632 | HSA10 | T | C | 29 | 0 | 29 | 0 | 0 | ***** |
| 2524093 | ICP5014 | G | A | 36 | 36 | 0 | 0 | 0 | ***** |
| 2557946 | ICP5011 | G | A | 34 | 34 | 0 | 0 | 0 | ***** |
| 2563779 | HGSA942 | C | A | 13 | 13 | 0 | 0 | 0 | ***** |
| 2568706 | HU106 | G | A | 15 | 15 | 0 | 0 | 0 | ***** |
| 2583083 | GRE108 | T | A | 18 | 18 | 0 | 0 | 0 | ***** |
| 2583279 | HU109 | C | T | 22 | 0 | 0 | 0 | 22 | ***** |
| 2600801 | S93 | T | A | 22 | 22 | 0 | 0 | 0 | ***** |
| 2605619 | FFP103 | A | C | 15 | 0 | 15 | 0 | 0 | ***** |
| 2614804 | HU25 | G | A | 20 | 20 | 0 | 0 | 0 | ***** |
| 2621386 | R35 | T | C | 26 | 0 | 26 | 0 | 0 | ***** |
| 2629515 | HU106 | C | A | 18 | 18 | 0 | 0 | 0 | ***** |
| 2691217 | S87 | A | G | 13 | 0 | 0 | 13 | 0 | ***** |
| 2694326 | HUSA304 | T | C | 18 | 0 | 18 | 0 | 0 | ***** |
| 2710989 | ICP5011 | A | G | 18 | 0 | 0 | 18 | 0 | ***** |
| 2736137 | ICP5062 | C | A | 12 | 12 | 0 | 0 | 0 | ***** |
| 2740206 | GRE4 | G | A | 17 | 17 | 0 | 0 | 0 | ***** |
| 2740236 | S7 | G | A | 29 | 29 | 0 | 0 | 0 | ***** |
| 2775023 | URU110 | T | C | 22 | 0 | 22 | 0 | 0 | ***** |
| 2775077 | AGT1 | C | T | 12 | 0 | 0 | 0 | 12 | ***** |
| 2775100 | S7 | T | C | 25 | 0 | 25 | 0 | 0 | ***** |
| 2797965 | CHI61 | A | G | 29 | 0 | 0 | 29 | 0 | ***** |
| 2827928 | CHL1 | A | G | 33 | 0 | 0 | 33 | 0 | ***** |
| 2831498 | 2A8 | A | C | 19 | 0 | 19 | 0 | 0 | ***** |
| 2842049 | 2A8 | C | T | 16 | 0 | 0 | 0 | 16 | ***** |
| 2864577 | HU106 | C | A | 17 | 17 | 0 | 0 | 0 | ***** |
| 2928262 | AGT9 | C | T | 14 | 0 | 0 | 0 | 14 | ***** |
| 2944648 | ICP5011 | T | C | 21 | 0 | 21 | 0 | 0 | ***** |
| 2950476 | HUSA304 | T | C | 17 | 0 | 17 | 0 | 0 | ***** |
| 2953174 | S25 | G | A | 24 | 21 | 0 | 3 | 0 | ***** |
| 2953210 | GRE4 | A | T | 14 | 0 | 0 | 0 | 14 | ***** |
| 2953225 | GRE317 | G | T | 15 | 0 | 0 | 0 | 15 | ***** |
| 2953227 | TUR9 | C | A | 14 | 14 | 0 | 0 | 0 | ***** |
| 2967947 | HU109 | T | C | 24 | 0 | 24 | 0 | 0 | ***** |
| 3016315 | S25 | G | A | 29 | 29 | 0 | 0 | 0 | ***** |
| 3033800 | BK2491 | G | A | 18 | 18 | 0 | 0 | 0 | ***** |
| 3037257 | CHL1 | G | A | 21 | 21 | 0 | 0 | 0 | ***** |
