# Supplementary material for: Atypical AT Skew in Firmicute Genomes Results from Selection and Not from Mutation
Source: PLoS Genet. 2011 Sep 15;7(9):e1002283. doi: 10.1371/journal.pgen.1002283 (PMC3174206; doi:10.1371/journal.pgen.1002283)
Supplement: Supplementary file 12 [file pgen.1002283.s009.doc]

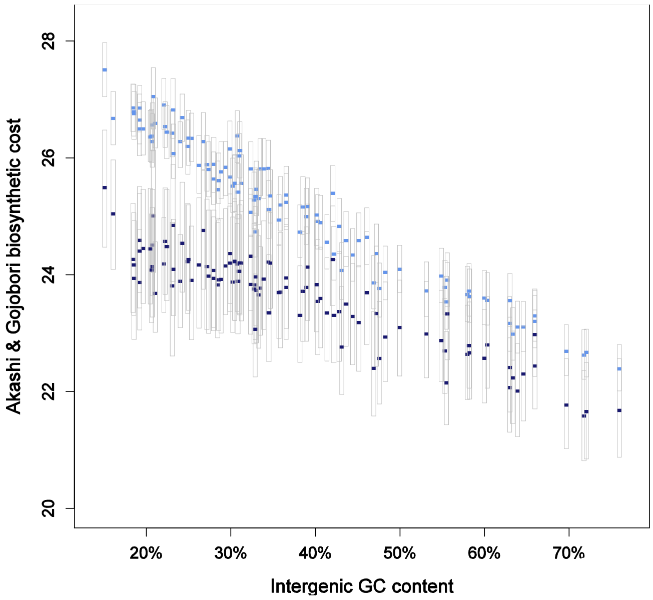
Akashi & Gojobori biosynthetic cost
28
26
24
22
20
20%
30%
40%
50%
60%
70%
Intergenic GC content

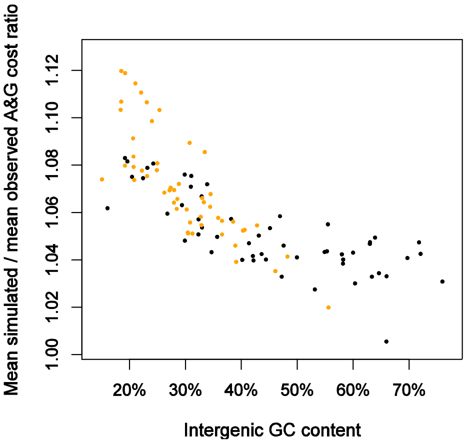
Mean simulated / mean observed A&G cost ratio
1.12
1.10
1.08
1.06
1.04
1.02
1.00
20%
30%
40%
50%
60%
70%
Intergenic GC content
