# Supplementary material for: Atypical AT Skew in Firmicute Genomes Results from Selection and Not from Mutation
Source: PLoS Genet. 2011 Sep 15;7(9):e1002283. doi: 10.1371/journal.pgen.1002283 (PMC3174206; doi:10.1371/journal.pgen.1002283)
Supplement: Supplementary file 13 [file pgen.1002283.s010.doc]

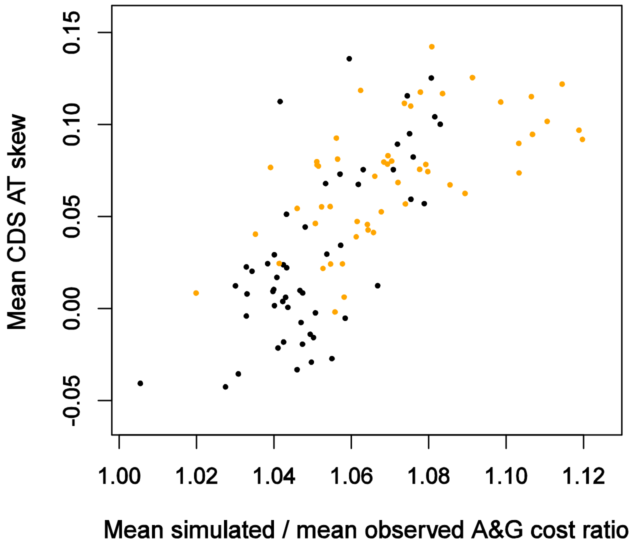

Mean CDS AT skew
-0.05
0.00
0.05
0.10
0.15
1.00
1.02
1.04
1.06
1.08
1.10
1.12
Mean simulated / mean observed A&G cost ratio
